# Divergent roles for Ly6C^+^CCR2^+^CX3CR1^+^ inflammatory monocytes during primary or secondary infection of the skin with the intra-phagosomal pathogen *Leishmania major*

**DOI:** 10.1371/journal.ppat.1006479

**Published:** 2017-06-30

**Authors:** Audrey Romano, Matheus B. H. Carneiro, Nicole A. Doria, Eric H. Roma, Flavia L. Ribeiro-Gomes, Ehud Inbar, Sang Hun Lee, Jonatan Mendez, Andrea Paun, David L. Sacks, Nathan C. Peters

**Affiliations:** 1 Laboratory of Parasitic Diseases, National Institute of Allergy and Infectious Diseases, National Institutes of Health, Bethesda, Maryland, United States; 2 Snyder Institute for Chronic Diseases, Departments of Microbiology Immunology and Infectious Diseases, Cumming School of Medicine, and Comparative Biology and Experimental Medicine, Faculty of Veterinary Medicine, University of Calgary, Calgary, AB, Canada; New York University, UNITED STATES

## Abstract

Inflammatory monocytes can be manipulated by environmental cues to perform multiple functions. To define the role of monocytes during primary or secondary infection with an intra-phagosomal pathogen we employed *Leishmania major*-red fluorescent protein (RFP) parasites and multi-color flow cytometry to define and enumerate infected and uninfected inflammatory cells in the skin. During primary infection, infected monocytes had altered maturation and were the initial mononuclear host cell for parasite replication. In contrast, at a distal site of secondary infection in mice with a healed but persistent primary infection, this same population rapidly produced inducible nitric oxide synthase (iNOS) in an IFN-γ dependent manner and was critical for parasite killing. Maturation to a dendritic cell-like phenotype was not required for monocyte iNOS-production, and enhanced monocyte recruitment correlated with IFN-γ dependent *cxcl10* expression. In contrast, neutrophils appeared to be a safe haven for parasites in both primary and secondary sites. Thus, inflammatory monocytes play divergent roles during primary versus secondary infection with an intra-phagosomal pathogen.

## Author summary

Many infectious diseases are initiated in the context of inflammation. This inflammatory response may be initiated by the pathogen itself or by damage to barrier sites associated with the infectious process. In the case of the vector-transmitted intra-phagosomal pathogen *Leishmania*, the parasite must contend with the robust inflammatory response initiated by the bite of an infected sand fly. Traditionally, rapid infection of macrophages in the skin and manipulation of these cells was seen as the mechanism by which the parasite avoided elimination by inflammatory cells. In the present study, we find that this is not the case following primary infection. After transient residence in neutrophils, *Leishmania* parasites transitioned into immature inflammatory monocytes, where they underwent proliferation and suppressed the maturation of these cells. In stark contrast, in a setting of pre-existing immunity, inoculation of parasites at a secondary site of infection resulted in parasite killing by monocytes in an IFN-γ dependent manner. Therefore, the role of monocytes is dependent upon the primary or secondary nature of the infection site into which they are recruited, emphasizing both the plasticity of this cell population and the central role these cells play during Leishmaniasis.

## Introduction

Immature bone marrow-derived monocytes are cells of the innate immune system that undergo maturation to populate numerous peripheral cell subsets [1–4]. Under different inflammatory and steady state conditions monocytes have been shown to acquire effector [5], regulatory [6], suppressor [7], homeostatic [1] or repair functions [8,9] and can also prime T helper 1 (Th1) adaptive immunity [10,11]. The recruitment of monocytes to sites of inflammation, their immature phenotype, and their plasticity suggests that these cells may be targets for infection and modulation by intra-phagosomal pathogens, such as *Mycobacteria tuberculosis*, *Cryptococcus neoformans*, *Salmonella enterica*, and *Leishmania spp*. [12].

Infection of mice with *Leishmania major (L*. *major)* is an established model to study inflammation and infection in the skin [13,14]. In nature, disease occurs when infected sand flies deposit parasites into the skin of a mammalian host during blood feeding, a process associated with significant tissue damage and inflammatory cell recruitment that is independent of the presence of the parasite. Once in the skin, *Leishmania* are predominantly engulfed by neutrophils, but are not killed. After 24–48 hours parasites transition into poorly defined CD11b^+^ mononuclear phagocytes, where they proliferate [15–17]. IFN-γ-producing T helper 1 (Th1) CD4^+^ T cells mediate protective immunity against *Leishmania* infection by activating infected cells to produce nitric oxide (NO) and kill parasites [18,19]. Healed but persistent primary *Leishmania* infection, in which viable parasites are maintained at low levels for the life of an infected individual, mediates rapid immunity at a distal site of secondary challenge and is the gold standard of protective immunity in both mice and people [20–22]. Understanding the nature of this immunity is critical to developing an effective *Leishmania* vaccine. Remarkably, neither the phagocytic cell that mediates parasite killing during secondary challenge, nor the phenotype of the secondary host cell during acute primary infection, have been carefully defined in the skin. Rather, previous work has focused on the role of monocyte-derived cells late in primary infection, and/or employed an inadequate set of phenotypic markers at acute time points [5,10,11,15,17,22]. It is not known if the phenotype or effector function of infected inflammatory cells during primary or secondary infection differ, and whether or not this is related to infection outcome.

In this study, we employed intra-dermal inoculation of *L*. *major*-RFP parasites and multicolor flow cytometry to track the fate of *L*. *major* and identify divergent roles for inflammatory monocytes during primary or secondary infection. These studies identify a critical early window during which protective immunity must act to prevent monocyte modulation and parasite expansion.

## Results

### *L*. *major* transitions from neutrophils to inflammatory monocytes, not tissue resident cells, during acute infection

**Fig 1:**
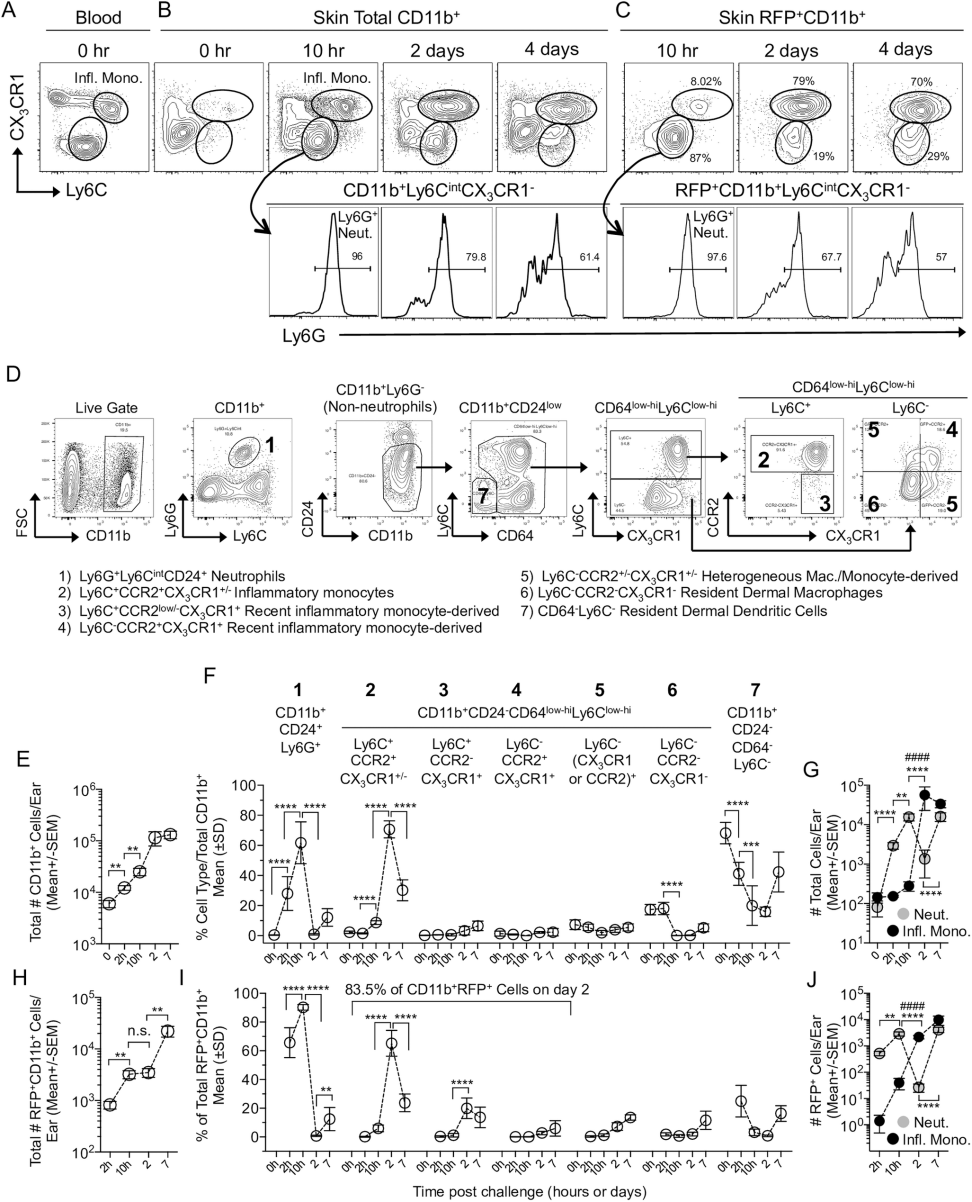
*L*. *major* transitions from neutrophils to inflammatory monocytes during acute infection. C57BL/6 CX_3_CR1-gfp mice were injected with 2x10^5^ *L*. *major*-RFP parasites i.d. in the ear. **(A-D)** Representative plots of blood or skin cells stained with the indicated markers. **(A-C)** CX_3_CR1 and Ly6C expression on total (A and B) or RFP^+^ (C) CD11b^+^ cells including Ly6G expression on the CX_3_CR1^-^Ly6C^int^ population (lower panels). Mono.: Inflammatory Monocytes; Neut.: Neutrophils. **(D)** Representative plots of Live cells (gates as per S1A Fig) from the skin on day 3 p.i.. (**E-J)** Kinetic analysis of total (E-G) or RFP^+^ (H-J) CD11b^+^ populations in the skin. **(E, G, H, and I)** Enumeration of cells using counting beads. **(F and I)** Frequency of the indicated subsets within total CD11b^+^ (F) or RFP^+^CD11b^+^ (I) cells. **(G and J)** Total (G) or RFP^+^ (J) number of neutrophils (Neut.) or Ly6C^+^CCR2^+^CX3CR1^+/-^ inflammatory monocytes (Infl. Mono.) per ear. Data are from one experiment representative of more than 3 similar experiments. Data are the mean. n = 4 (0h), 6 (2h, 10h, 2d) or 10 (7d) ears per group. In E, F, H, and I the asterisks indicate a significant difference between the time points referred to by the brackets. In G and J the symbols indicate a significant difference between the numbers of neutrophils (*) or monocytes (#) at the time points referred to by the brackets. The level of significance is indicated in the materials and methods.

In order to define the phenotype of inflammatory cells during the transition of the *L*. *major* parasite from neutrophils to secondary phagocytic cells between approximately 1 and 4 days we initially employed CX_3_CR1^+/gfp^ reporter mice (Fig 1A–1C, and S1A Fig-gating strategy); [15,23]. Prior to challenge, CD11b^+^Ly6C^hi^CX_3_CR1^+^ inflammatory monocytes were abundant in the blood but rare in the skin (Fig 1A versus 1B, 0hr.). Following needle inoculation of *L*. *major*-RFP, CD11b^+^Ly6C^hi^CX_3_CR1^+^ inflammatory monocytes and Ly6C^int^CX_3_CR1^-^Ly6G^+^ neutrophils infiltrated the dermal site of infection (Fig 1B). Ly6C clearly defined inflammatory monocytes at 10 hours post infection (p.i.) and these cells co-expressed CCR2 but were MHCII^-^ and CD11c^lo^ (S1B Fig). Analysis of the cellular infiltrate following exposure to sand fly bites, the natural mode of *Leishmania* inoculation, also revealed the robust infiltration of Ly6C^hi^ inflammatory monocytes and neutrophils (S2 Fig). Of note, this robust recruitment following exposure to sand fly bites is not dependent upon the parasite or pre-existing immunity. At 2 and 4 days p.i., CX_3_CR1 was required to definitively track the CD11b^+^Ly6C^+^CX_3_CR1^+^ inflammatory monocyte population due to decreased expression levels of Ly6C (Fig 1B; Ly6C MFI, 10hr 12634 +/-SD 1891 versus 2 days 4071 +/-SD 600; p<0.0001, n = 6). At 10h p.i. the vast majority of RFP^+^ infected cells (S1C Fig) were neutrophils, as previously shown (Fig 1C; [15]). However, at 2 days p.i., RFP^+^ cells were largely Ly6C^int/hi^CX_3_CR1^+^, strongly suggesting a transition of the majority of parasites into inflammatory monocytes. A similar pattern was observed at 4 days p.i..

**Fig 2:**
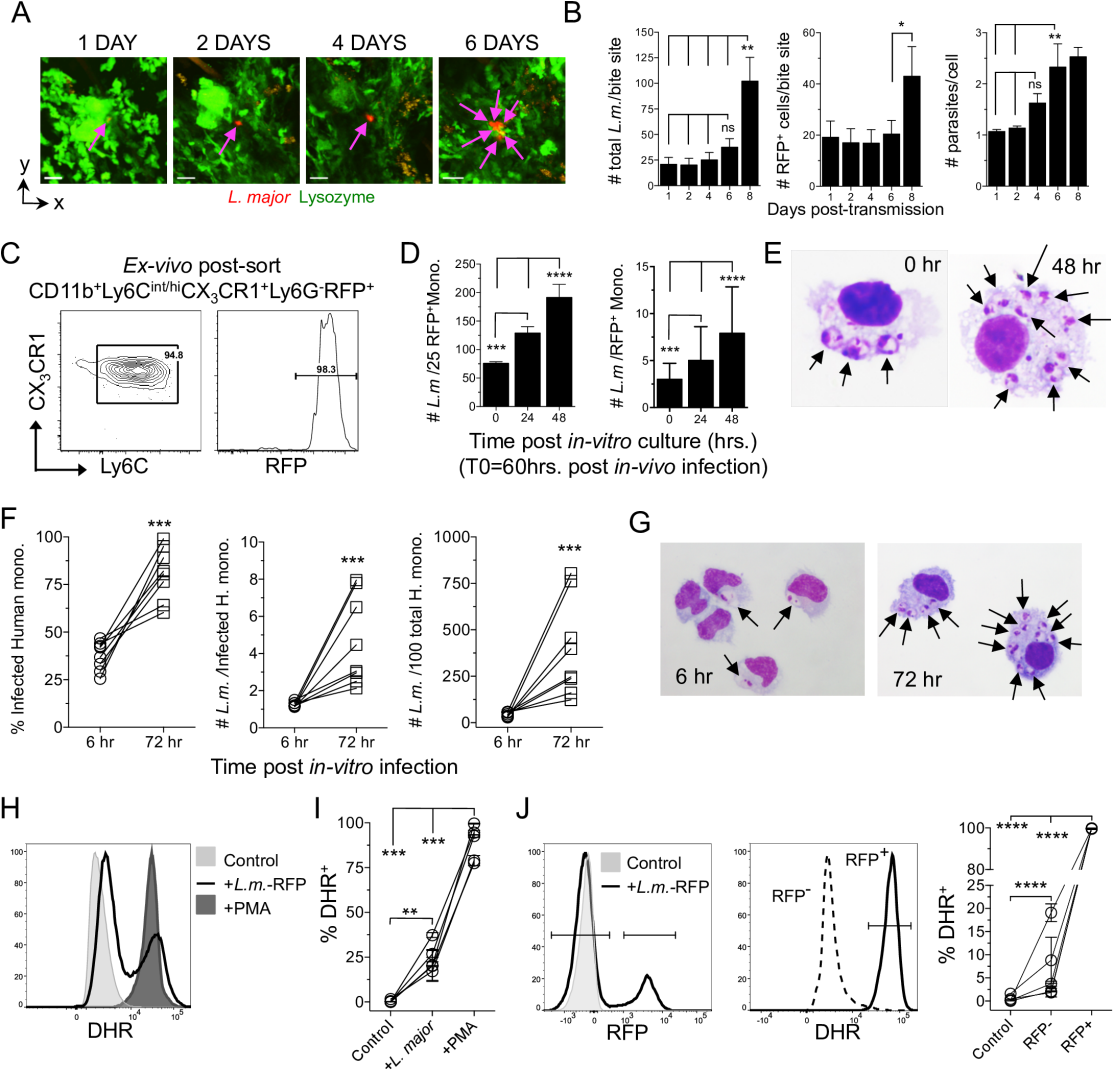
*L*. *major* undergoes delayed proliferation until transitioning into monocytes that are permissive for parasite replication. **(A and B)** LysM-gfp mice were exposed to the bites of *L*. *major-*RFP infected sand flies and individual inoculation sites were sequentially imaged employing 2P-IVM. **(A)** Representative images from the same bite site of a putative single infected cell from 1 to 6 days post-bite. **(B)** Enumeration of the number of parasites and infected cells (parasites are assumed to be intracellular) per individual bite site employing slice-by-slice visual analysis of the z-stack to determine parasite and infected cell number. Data are the Mean +/- SEM, n = 7 bite sites. **(C-E)** CX_3_CR1-gfp mice were needle inoculated with 2x10^5^ *L*. *major*-RFP parasites in the ear. Sixty hours post-infection CD11b^+^Ly6C^int/hi^CX_3_CR1^+^Ly6G^-^RFP^+^ cells were cell-sorted and placed in *in-vitro* culture. **(C)** Post-sort analysis of the RFP^+^ sorted population. **(D)** Quantitative cytospin analysis of the total number of amastigotes per 25 RFP^+^ monocytes (left panel; 100 total monocytes per time point, a denominator of 25 was chosen arbitrarily) or the number of amastigotes per individual monocyte (right panel; n = 100 (d0); 123 (24h) or 179 (48 h), total monocytes). Data are the mean +/- SD. Data are from one experiment representative of 3 similar experiments. **(E)** Representative cytospin images at 0h and 48h of RFP^+^ murine monocytes. **(F-J)** *In-vitro L*. *major* infection of human elutriated monocytes. **(F)** Percent infected, number of parasites per each infected, and total number of *L*. *major* per 100 total, monocytes. Each data set is an individual experiment employing an individual donor (n = 8), employing ≥108 infected monocytes per time point per donor. **(G)** Representative cytospin images. **(H-J)** Respiratory burst in human monocytes employing DHR. **(H)** Representative histogram of DHR expression by total elutriated monocytes under the indicated conditions. **(I)** % DHR^+^ monocytes under the indicated conditions. **(J)** Monocytes were identified as uninfected or infected based upon RFP expression (left panel) and the frequency of DHR^+^ cells (middle panel) was determined (right panel). In (H-J) data are from 5 experiments employing 5 individual donors.

We next extended the set of phenotypic markers to definitively track inflammatory monocytes according to a gating strategy outlined in Fig 1D and based upon previous observations [3]. Kinetic analysis employing counting beads to enumerate the inflammatory cell infiltrate into the ear revealed the total number of CD11b^+^ cells per ear increased significantly by 2 and 10 hours p.i. (Fig 1E). This increase was attributable to CD11b^+^CD24^+^Ly6G^+^ neutrophil recruitment, which represented an increasing proportion (Fig 1F, Population 1) and number (Fig 1G, Neut.) of total CD11b^+^ cells in the skin, and corresponded with a decrease in the proportion of CD64^low-hi^Ly6C^-^CCR2^-^CX_3_CR1^-^ dermal macrophages (Population 6), and pre-existing CD64^-^Ly6C^-^ cells, that contain dermal DCs (Population 7) (Fig 1F). Following the initial recruitment of neutrophils, when neutrophils significantly outnumbered monocytes (Fig 1G, 2h and 10h, p≤0.0001, n = 6), inflammatory monocytes, defined as CD11b^+^CD24^-^CD64^low-hi^Ly6C^+^CCR2^+^ cells, which are virtually all CX_3_CR1^+^ (Fig 1D, Population 2), were recruited into the skin (Fig 1F and 1G), likely from the blood (S3A Fig) where they pre-exist as MHCII^-^CD11c^-^ cells (S3B Fig). These cells increased dramatically in proportion (Fig 1F) and number (Fig 1G) between 10h and 2d post-infection and significantly outnumbered neutrophils on day 2 p.i. (Fig 1G; p≤0.0001, n = 6). Kinetic analysis of the total number of RFP^+^ infected cells (S1C Fig and S3C Fig) revealed an increase in infected cell numbers due to neutrophil uptake of inoculated parasites at 2 and 10h p.i. (Fig 1H and 1I; [15,17]). Between 10h and 2 days the phenotype of infected cells transitioned dramatically from 90.2% (+/-SD 2.4, n = 6) neutrophils to 83.5% (+/-SD 1.1, n = 6) inflammatory monocytes or putative inflammatory monocyte derived cells (Defined as Ly6C^+^CCR2^low/-^CX_3_CR1^+^, Population 3, or Ly6C^-^CCR2^+^CX_3_CR1^+^, Population 4, cells) (Fig 1I), without a significant change in the total number of infected cells (Fig 1H). This transition was also reflected in the absolute number of infected inflammatory cells (Fig 1J), where RFP^+^ neutrophils significantly outnumbered RFP^+^ inflammatory monocytes at 10h (p<0.0001, n = 6), but then dropped dramatically on day 2 p.i., when the number of infected monocytes underwent a corresponding increase and significantly outnumbered infected neutrophils (p = 0.0003, n = 6). Importantly, there was no significant difference (p = 0.80, n = 6) in the number of infected neutrophils at 10 hours p.i. (Population 1: Mean 2890 +/-SEM 532.2, n = 6) versus the number of infected inflammatory monocytes or recent inflammatory monocyte derived cells at 2 days p.i. (Populations 2, 3, and 4: Mean 2718 +/-SEM 411, n = 6). This data suggests that *L*. *major* transitions from neutrophils to inflammatory monocytes without significant parasite expansion or elimination as determined by the number of infected cells.

### Murine and human monocytes are a permissive host cell for *L*. *major* during acute infection

Infected neutrophils do not kill *L*. *major* at acute time points p.i. and inoculation of these infected neutrophils efficiently initiates disease [15]. In contrast, monocytes have been implicated in parasite killing following primary *L*. *major* infection of the intra-peritoneal (i.p.) cavity [24]. To determine the fate of parasites during the transition phase we first employed 2-photon intra-vital microscopy (2P-IVM) to sequentially image the same site of *L*. *major-*RFP deposition in the skin following exposure to infected sand fly bites (Fig 2A). Enumeration of RFP^+^ parasites employing a slice-by-slice visual analysis of the image z-stack revealed the total number of parasites per bite site and the number of infected cells did not significantly change until day 8 post-transmission, when it increased (Fig 2B). In addition, the number of parasites per cell did not significantly change until day 6 of infection (Fig 2A and 2B, right panel), which is consistent with a lack of parasite killing as well as a delay in parasite proliferation during neutrophil to monocyte transition.

We also sorted infected CD11b^+^Ly6C^int/hi^CX_3_CR1^+^Ly6G^-^RFP^+^ inflammatory monocytes from the skin 60h post *in-vivo*-infection (Fig 2C) and assayed parasite growth *in-vitro*. Cell sorting did not employ CCR2, as optimal CCR2 staining required incubation at 37°C that reduced the yield of infected cells. Analysis of the total number of intra-cellular parasites per 25 RFP^+^ monocytes and the number of parasites per monocyte showed significant proliferation at 24 and 48h of *in-vitro* culture, demonstrating these cells are permissive host cells for *L*. *major* (Fig 2D and 2E). To ensure our observations were not specific to murine monocytes we also infected elutriated human monocytes (S4 Fig) *in-vitro*. We observed robust expansion of *L*. *major* between 6 and 72h as assessed by the number of infected cells, parasites per single infected cell, and parasites per 100 total cells (Fig 2F and 2G). Parasites grew despite triggering respiratory burst as assessed by Dihydrorhodamine 123 (DHR) (Fig 2H and 2I). Remarkably, virtually every infected monocyte was positive for DHR (Fig 2J) suggesting these cells are activated, but fail to kill *L*. *major*. The ability of *L*. *major* to survive respiratory burst may be due to the fact that ROS have been shown to be excluded from parasite containing phagolysosomes in infected cells [25]. Therefore, inflammatory monocytes serve as a permissive host cell during acute primary infection in the skin.

### Disparate inflammatory monocyte responses at sites of primary and secondary infection

**Fig 3:**
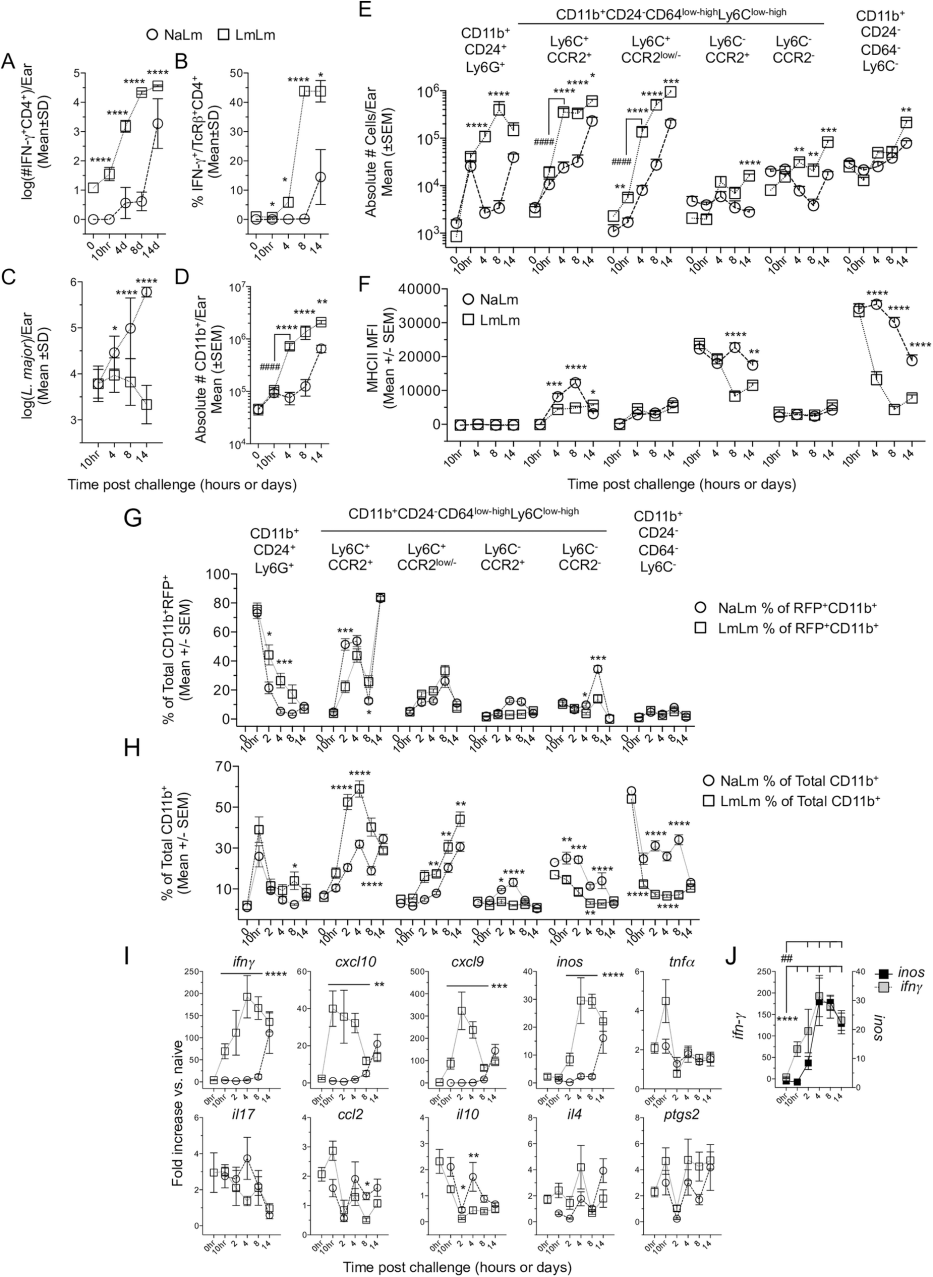
Disparate inflammatory monocyte responses at sites of primary and secondary infection. Naïve wild type B6 mice (NaLm) or mice with a healed but persistent primary infection (LmLm) were challenged in the ear with 2x10^5^ *L*. *major* (A-F) or *L*. *major*-RFP (G-J). At the indicated times p.i. the ear dermis was analyzed as indicated. 0h time point data are prior to challenge. Statistical analysis refers to comparison between NaLm and LmLm groups (asterisks) or between time points (#). **(A)** The number of IFN-γ^+^CD4^+^ T cells/ear determined by multiplying the absolute number of CD3^+^CD4^+^ T cells per ear employing counting beads by the frequency of cells with the capacity to make IFN-γ following APC+*L*. *major*-Ag re-stimulation. **(B)** The frequency of cells with the capacity to make IFN-γ following APC+*L*. *major*-Ag re-stimulation. **(C)** The number of parasites per ear as determined by LDA. **(D)** The absolute number of CD11b^+^ cells per ear as determined employing counting beads. **(E)** The total number of the indicated populations per ear during primary (NaLm) or secondary (LmLm) infection. Cells were gated as generally described in S3 Fig. **(F)** Median fluorescence intensity of MHCII on the indicated populations over time. Data in (A-F) are from one experiment representing more than 3 similar experiments. Data are the Mean +/- error as indicated in the figure, n = 7–8 ears per time point. **(G and H)** Analysis of the frequency of the indicated populations within the infected RFP^+^CD11b^+^ (G) or total CD11b^+^ (H) population. **(I)** mRNA expression from the ears depicted in G and H, the values shown are the fold increase relative to expression in naive ears. Data are the mean +/- SEM. Data are pooled from two similar experiments. In (I) statistical analysis refers to comparison between NaLm and LmLm groups: *ifn*γ p<0.0001 on 10hr, d2, d4 and d8; *cxcl10* p≤0.005 on 10hr, d2, d4, and d8; *cxcl9* p≤0.0005 on 10hr, d2, d4, and d8; *inos* p<0.0001 on d2, d4, d8; *ccl2* p = 0.0483 on d8; il10 *, p = 0.036, **, p = 0.004. Line refers to time points that were statistically significant. **(J)** Synchronous comparison of the *ifn*γ and *inos* expression kinetic taken from (I). ##, p≤0.0041 comparing *inos* 0hr with d2, 4, 8, and 14. ****, p≤0.0001 comparing *ifn*γ 0hr with 10hr, d2, 4, 8, and 14.

We next turned our attention to the role of inflammatory monocytes at secondary sites of infection. We hypothesized that rapid activation of infected immature inflammatory monocytes during neutrophil to monocyte transition is the effector mechanism by which healed but persistent primary infection mediates protective Th1 immunity [20,22]. Mice with a healed but persistent primary *Leishmania* infection where generated by inoculation of 10^4^ *L*. *major* metacyclic promastigotes into the footpad [26]. As expected, mice with a persistent primary infection mounted a robust Th1 immune response at a secondary site of challenge in the ear (LmLm group) (Fig 3A and 3B), coincident with significant control of parasite numbers versus mice with a primary infection (NaLm group) starting on day 4 (Fig 3C). Evaluation of the absolute number of cells per ear revealed no difference in the total number of CD11b^+^ cells in LmLm versus NaLm mice at 10h post-challenge (post-ch) (Fig 3D), and no difference in the number of neutrophils (Fig 3E, left side), suggesting the acute neutrophil response at primary or secondary sites of infection is similar. In stark contrast, LmLm mice had a dramatic almost 10-fold increase in the number of CD11b^+^ cells on day 4 post-ch, correlating with the recruitment of IFN-γ^+^CD4^+^ T-cells into the ear (Compare Fig 3D and 3A). This increase was attributable to huge increases in the number of inflammatory monocytes and CCR2^low/-^ recent inflammatory monocyte derived cells (Fig 3E). These differences were not due to an alteration in monocyte frequencies in the blood of naïve animals (NaNa) versus those with a healed lesion but persistent parasites (LmNa) prior to challenge (S5A Fig). Neutrophil numbers were also maintained at secondary versus primary sites on day 4 and 8 post-ch. (Fig 3E). Despite large numbers of IFN-γ producing CD4^+^ T cells in the skin, monocytes and monocyte derived cells had lower levels of MHCII expression at the population level during secondary infection compared to primary infection (Fig 3F). This is likely due to the large and sustained numbers of infiltrating immature monocytes during secondary infection (Fig 3E).

Analysis of RFP^+^ infected cells during primary (NaLm) or secondary (LmLm) infection revealed NaLm mice had a faster transition of parasites from neutrophils to Ly6C^+^CCR2^+^ inflammatory monocytes between 10h and 2d (Fig 3G) as indicated by higher frequencies of infected monocytes on day 2. This occurred despite a smaller proportion of inflammatory monocytes within the total CD11b^+^ population versus LmLm mice (Fig 3H). However, by day 4 the frequency of total infected cells that were inflammatory monocytes was similar in both settings (Fig 3G). While RFP expression does not definitively assess parasite viability, increased frequencies of RFP^+^ neutrophils at secondary versus primary sites at 2 and 4 days post-challenge suggests these cells are acting as a safe haven for parasites during secondary infection (Fig 3G) [15].

We also analyzed gene expression by RT-qPCR over time in NaLm and LmLm mice. As expected we saw an early *ifn*γ response at 10h post-ch in LmLm but not NaLm mice (Fig 3I) and this was associated with increased levels of the chemokines *cxcl10* and *cxcl9*, both of which are induced by exposure to IFN-γ, and in the case of CXCL10, is involved in the recruitment of monocytes [27,28]. Peak *cxcl10* expression occurred at 10h p.i., correlating with subsequent monocyte recruitment. We also assessed *inducible-nitric oxide synthase (inos)* expression, the enzyme responsible for NO production. We found *inos* expression was not significantly upregulated until day 2 post-challenge in LmLm versus NaLm mice, suggesting the up regulation of *inos* expression is delayed until after the recruitment of IFN-γ producing CD4 T cells (Fig 3I and 3J). In contrast, expression of *tnfa*, *il17*, *ccl2*, *il10*, *il4*, and *ptgs2*, were not significantly different in NaLm versus LmLm mice. In addition, the low levels of *ccl2* expression strongly suggest that the IFN-γ inducible chemokines CXCL9 and specifically CXCL10, is the primary driver of monocyte recruitment at the secondary site of challenge. Therefore, at a site of secondary challenge inflammatory monocyte recruitment and infection correlated with parasite killing at day 4 p.i..

### Divergent inflammatory monocyte phenotypes during primary versus secondary infection

**Fig 4:**
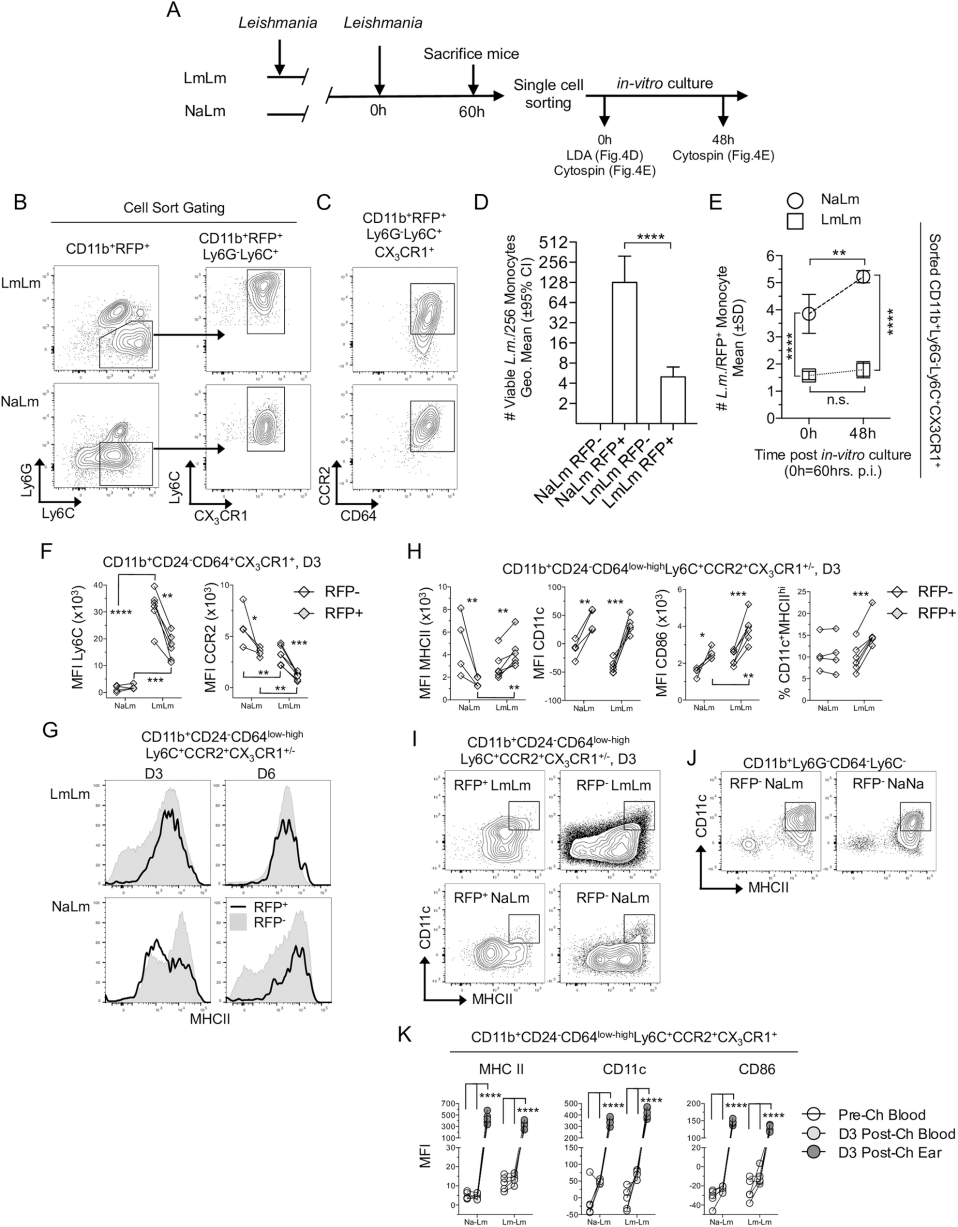
Inflammatory monocytes are activated and mediate parasite killing at sites of secondary infection. Naïve CX_3_CR1-gfp mice (NaLm) or mice with a chronic primary infection (LmLm) were challenged in the ear with 2x10^5^ *L. major*-RFP. **(A)** Schematic of experiments performed in (B-E). **(B)** Representative gating strategy for sorting CD11b^+^RFP^+^Ly6G^-^Ly6C^+^CX_3_CR1^+^ cells from NaLm or LmLm mice on day 3 (60h) post-ch. Post-sort analysis is shown in Fig 2C. **(C)** Representative CCR2 and CD64 expression on CD11b^+^RFP^+^Ly6G^-^Ly6C^+^CX_3_CR1^+^ cells. **(D)** Two-fold limiting dilution analysis to determine the number of monocytes containing at least one viable parasite from 256 infected (RFP^+^) or uninfected (RFP^-^) cells sorted and plated at 60 hrs. post-ch. and analyzed at 7–10 days post *in-vitro* culture. Data are the Geometric mean +/- 95% CI of the last positive well of 4 replicates employing 256 cells per well. Data are from one experiment representing 3 repeat experiments. **(E)** *Ex-vivo* quantitative cytospin analysis of the total number of amastigotes per RFP^+^ monocyte. Data are the Mean+/-SD pooled from two experiments employing n = 108 (NaLm 48h), 320 (NaLm 0h), 323 (LmLm 0h) or 608 (LmLm 48h) total RFP^+^ monocytes. **(F-J)** Analysis of cell surface expression of the indication markers on RFP^+^ (closed diamonds) or RFP^-^ (open diamonds) monocytes from the same ear on day 3 p.i.. **(F)** Ly6C and CCR2 MFI. **(G)** Representative MHC II histograms. **(H)** MHCII, CD11c, CD86 MFI, and % of CD11c^+^MHCII^hi^ cells within the indicated population. **(I)** Representative plots of MHCII and CD11c expression of the population described in (G) and (H). The CD11c^+^MHCII^hi^ dendritic cell gate is indicated based on the dermal DC population as indicated in **(J)** and is the same in each panel. Data are from one experiment representing more than three similar experiments. **(K)** Analysis of MHC II, CD11c and CD86 MFI on monocytes as defined in (G) and (H) in the blood or ear at the indicated times prior to or following challenge. Results are representative of two similar experiments.

To determine the fate of parasites that transitioned into inflammatory monocytes during primary versus secondary infection we performed experiments as outline in Fig 4A. Infected and uninfected monocytes were sorted from ear skin 60h post *in-vivo*-infection as described in Fig 2E (Fig 4B). Infected CD11b^+^Ly6C^int/hi^CX_3_CR1^+^Ly6G^-^RFP^+^ inflammatory monocytes were virtually all CD64^+^ and CCR2^int-high^ (Fig 4C). Sorted RFP^-^ and RFP^+^ monocytes from NaLm or LmLm mice were then subjected to limiting dilution analysis, in which a known number of cells underwent 2-fold dilution to determine the number of cells containing at least one viable parasite that was able to grow after 7–10 days of *in-vitro* culture in the titration plate. Our analysis revealed there was no difference between the number of RFP^+^ monocytes from NaLm mice with at least one viable parasite versus the 256 RFP^+^ cells plated in the assay (p = 0.055, n = 4). In contrast, only approximately 4 of the 256 RFP^+^ monocytes from LmLm mice contained parasites capable of expanding *in-vitro* (Fig 4D). Cytospin analysis of sorted RFP^+^ monocytes from LmLm mice directly *ex-vivo* confirmed that these cells do contain intact parasites (Fig 4E, 0h time-point). However, unlike parasites from NaLm mice, these parasites fail to proliferate over a 48-hour period when plated *in-vitro* (Fig 4E). Therefore, inflammatory monocytes from secondary sites of infection are not a permissive host cell for *L*. *major*. Infected monocytes from LmLm mice also contained fewer parasites per infected cell (Fig 4E, 0h). This may be due to a lower *in-vivo* multiplicity of monocyte infection due to the slower transition of parasites into inflammatory monocytes at the 60h time point as suggested in Fig 3G. Alternatively, it may reflect increased parasite killing and/or suppression of parasite growth in LmLm mice prior to single cell sorting and analysis.

Previous studies suggest CCR2^+^ monocytes undergo maturation to become dendritic cells during late primary infection [5]. We measured Ly6C and CCR2 expression on CD11b^+^CD24^-^CD64^+^CX_3_CR1^+^ putative monocytes on day 3 post-ch to determine the influence of infection and pre-existing immunity on monocyte maturation (Fig 4F). Ly6C expression was much higher on cells from secondary versus primary sites of infection, likely due to the sustained recruitment of these cells in LmLm mice (Fig 3E), while CCR2 expression was lower. Comparison of RFP^+^ versus RFP^-^ cells revealed RFP^+^ infected cells had lower expression of Ly6C in LmLm mice, while in NaLm mice there was no change. CCR2 was reduced on RFP^+^ versus RFP^-^ cells from both NaLm and LmLm groups but this was significantly enhanced in LmLm mice (Fig 4F, right panel). Prior to challenge, these differences were not present on putative CX_3_CR1^+^ monocytes in the blood (S5B Fig). Therefore, pre-existing immunity and infection status alter the phenotype of the inflammatory CD64^low-high^CX3CR1^+^ monocyte population in the skin, with secondary sites of infection containing monocytes with higher Ly6C expression and lower CCR2 expression. The lower levels of Ly6C and CCR2 on RFP^+^ infected versus uninfected monocytes during secondary infection suggest these cells are undergoing maturation. Subsequent analysis of Ly6C^+^CCR2^+^CX_3_CR1^+/-^ monocytes on day 3 post-challenge, when monocytes represent the majority of infected mononuclear cells, revealed MHCII expression was significantly reduced on RFP^+^ versus RFP^-^ monocytes from NaLm mice but this difference was lost by day 6 (Fig 4G and 4H, far left panel), suggesting early modulation of monocyte maturation by *Leishmania* during acute primary infection. In contrast, RFP^+^ cells from LmLm mice had higher expression of MHCII versus RFP^-^ cells or RFP^+^ cells from NaLm mice (Fig 4H). CD11c and CD86 were increased on RFP^+^ cells in both NaLm and LmLm mice, however, CD86 was higher on RFP^+^ cells from LmLm mice, suggesting that infected monocytes at secondary sites of infection undergo enhanced maturation versus those at primary sites. While the frequency of monocytes that were CD11c^+^MHCII^hi^ monocyte derived dendritic cell was significantly higher among infected versus uninfected cells in LmLm mice (Fig 4H, far right panel and I), the vast majority (Mean 84.5% +/- SD 3.5% n = 6) had not matured to become CD11c^+^MHCII^hi^ DCs at this time-point as defined employing a DC gate based on CD11c and MHCII expression levels on dermal DCs in the CD64^-^Ly6C^-^ population (Fig 4J). Because Ly6C^+^CCR2^+^CX_3_CR1^+^ monocytes originate as MHCII^-^CD11c^-^CD86^-^ immature cells in the blood (Fig 4K; see also S5C Fig and S3C Fig), our data suggest that *L*. *major* actively prevents the maturation of inflammatory monocytes towards an MHCII^+^ phenotype at the site of infection, but that this does not occur at sites of secondary challenge.

### Inflammatory monocytes are the predominant iNOS^+^ cell type at sites of *Leishmania* challenge

**Fig 5:**
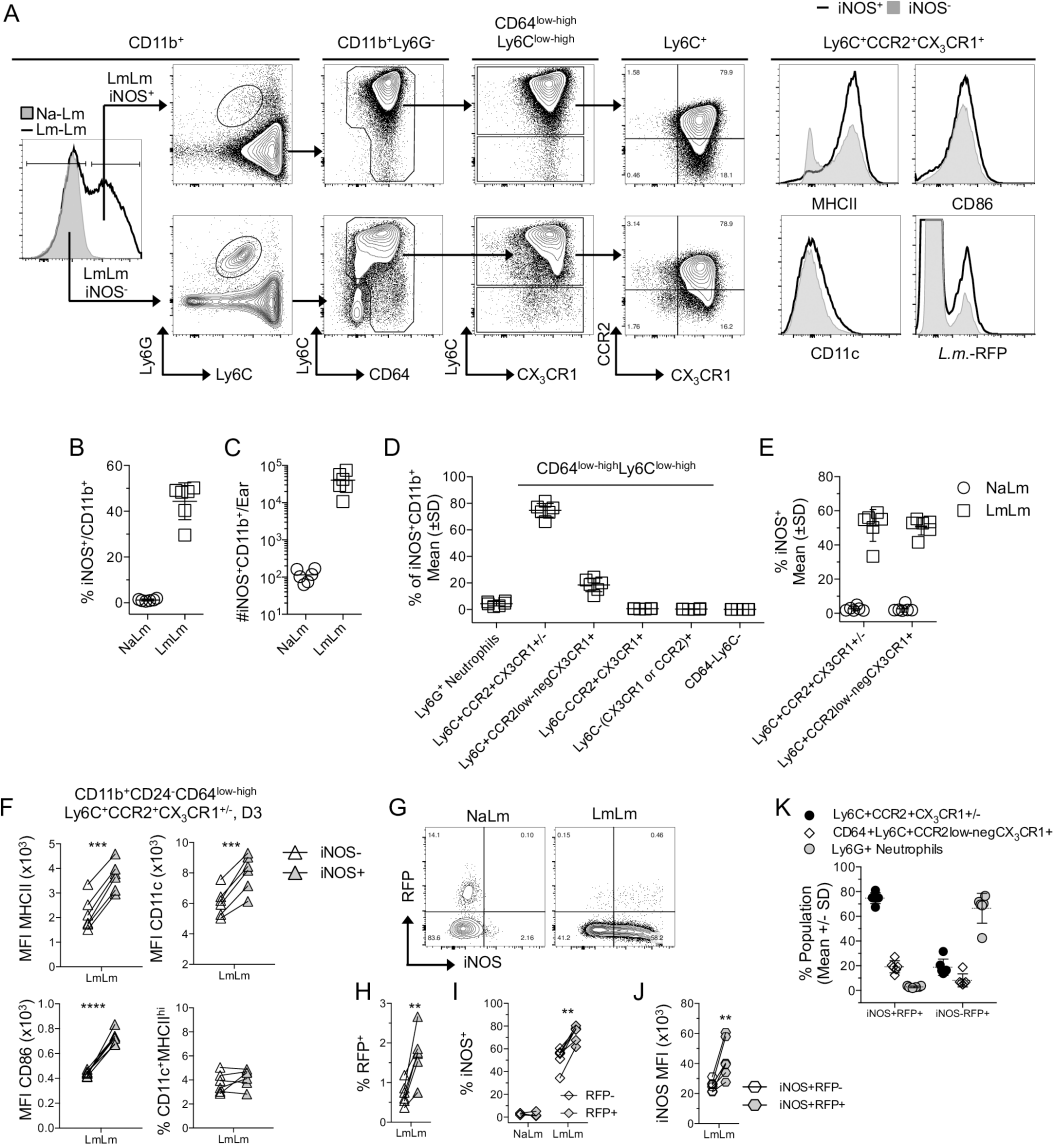
Immature inflammatory monocytes are the predominant iNOS^+^ cell type at sites of *L*. *major* challenge. Mice as described in Fig 4 were analyzed for iNOS expression on day 3 post-ch. with *L*. *major*-RFP. **(A)** Representative histogram and plots of iNOS^+^ or iNOS^-^ cells. **(B)** % or **(C)** absolute number of CD11b^+^ cells that are iNOS^+^ on day 3 post-ch. **(D)** Frequency of the indicated populations within the total iNOS^+^CD11b^+^ population. **(E)** Percentage of the indicated populations on the x-axis that are iNOS^+^. **(F)** MHCII, CD11c, CD86 MFI of, and % of CD11c^+^MHCII^hi^ cells within iNOS^+^ or iNOS^-^ monocytes obtained from the same site of infection. **(G)** Representative RFP and NOS2 staining of the population described in (F). **(H)** Percentage of iNOS^+^ or iNOS^-^ monocytes gated as in (F) that are RFP^+^. **(I)** Percentage of RFP^+^ or RFP^-^ monocytes gated as in (F) that are iNOS^+^. **(J)** iNOS MFI of iNOS-producing RFP^-^ versus RFP^+^ monocytes gated as in (F). Each paired data set represents cells from the same ear. **(K)** Percentage of the indicated populations within the total CD11b^+^ infected iNOS^+^ versus infected iNOS^-^ populations. Data are from one experiment representing 3 similar experiments.

We next wished to determine the phenotype of iNOS^+^ cells at sites of secondary challenge (Fig 5A). On day 3 post-ch, LmLm mice contained large numbers and frequencies of CD11b^+^iNOS^+^ cells (Fig 5A, left panel, and Fig 5B and 5C). Phenotypic analysis revealed that CD11b^+^iNOS^+^ cells were almost exclusively inflammatory monocytes followed by a small proportion of CCR2^low-neg^ recent inflammatory monocyte derived cells (Fig 5D). Loss of CCR2 on CD64^low-high^Ly6C^hi^ monocytes was not associated with changes in the proportion of these cells that can make iNOS, suggesting maturation towards a CCR2^-^ phenotype was not a prerequisite for iNOS production (Fig 5E). iNOS^+^ inflammatory monocytes expressed higher levels of MHCII, CD11c, and CD86 versus iNOS^-^ cells (Fig 5F). However, iNOS production did not correlate with an increase in the proportion of cells that were CD11c^+^MHCII^hi^ DCs (Fig 5F, lower right). As expected, NaLm mice contained large frequencies of RFP^+^iNOS^-^ monocytes, while LmLm mice contained large numbers iNOS^+^ cells (Fig 5G). Cells that were iNOS^+^ were 2.4 times as likely to be RFP^+^ versus iNOS^-^ cells (Fig 5H) and, while RFP^-^ monocytes did contain high frequencies of iNOS^+^ cells, the RFP^+^ infected population contained significantly more (Fig 5I). Lastly, among iNOS^+^ monocytes, RFP^+^ cells had higher expression levels of iNOS versus RFP^-^ cells (Fig 5J), demonstrating that infected cells are both more likely to be iNOS^+^ and are activated to produce higher levels of iNOS. Therefore, iNOS production and infection are correlated. Of interest, analysis of the infected iNOS^-^ phagocyte population revealed that neutrophils were once again the predominant RFP^+^ cell type, suggesting neutrophils are acting as a safe haven during secondary infection (Fig 5K).

### Interferon-γ drives chemokine production, monocyte recruitment, iNOS production and parasite killing at sites of secondary challenge

**Fig 6:**
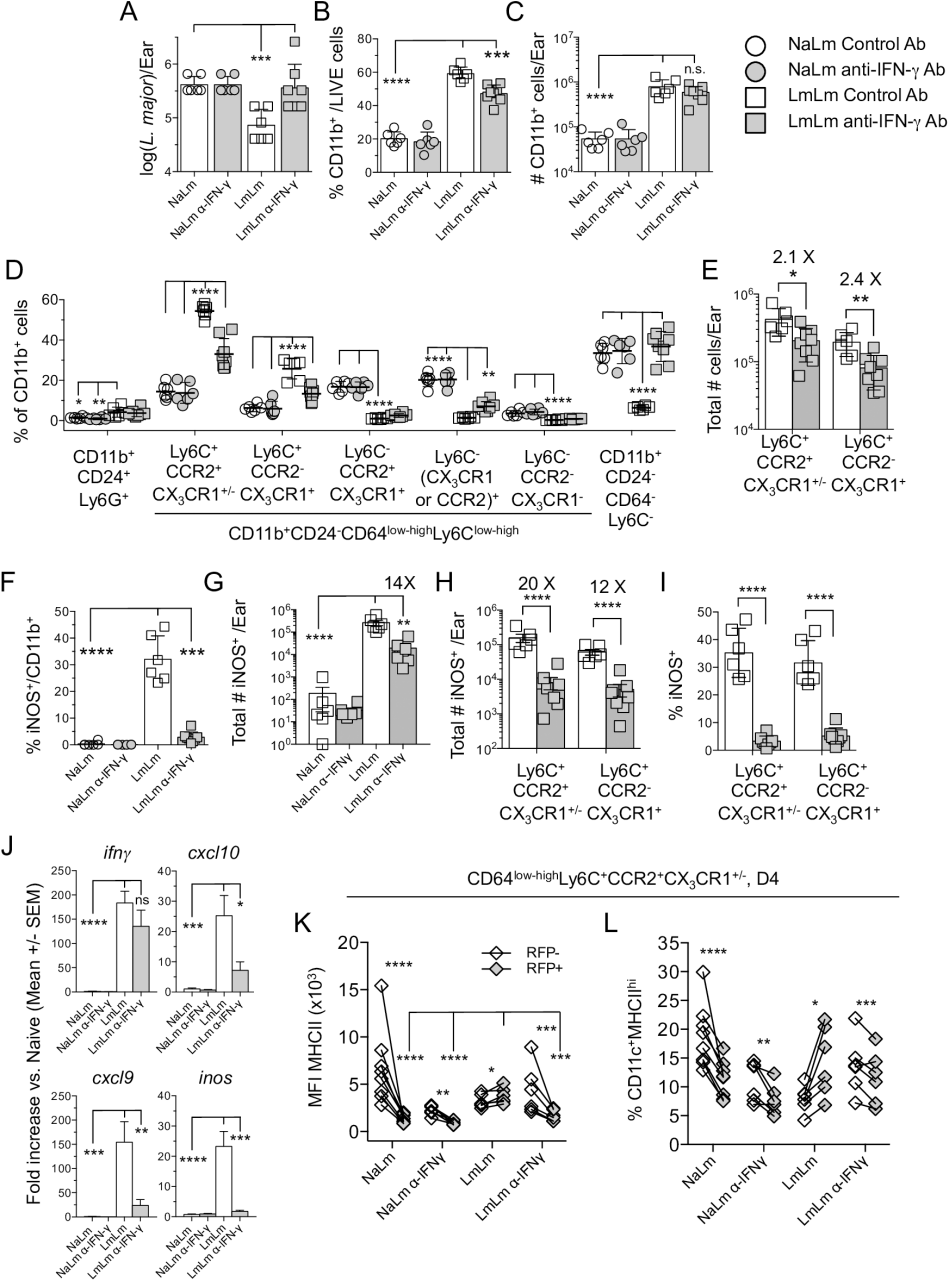
Interferon-γ drives chemokine expression, monocyte recruitment, iNOS-production and parasite killing at sites of secondary challenge. Mice as described in Fig 4 were treated with 0.5mg i.p. anti-IFN-γ or control antibody on day -1 and analyzed on day 4 post-ch. **(A)** Parasite load per ear determined by LDA. **(B and C)** Frequency (B) or absolute number (C) of CD11b^+^ cells per ear. **(D)** Frequency of the indicated populations within the total CD11b^+^ population per ear. **(E)** Absolute number of the indicated monocyte populations per ear. **(F and G)** Frequency of iNOS^+^ within total CD11b^+^ cells (F) and absolute number of CD11b^+^iNOS^+^ cells (G) per ear. **(H)** Absolute number of the indicated iNOS^+^ monocyte populations per ear. **(I)** Frequency of iNOS^+^ cells within the total indicated monocyte populations. **(J)** Gene expression was quantified as reported in Fig 3J. **(K)** MHCII MFI of RFP^+^ or RFP^-^ monocytes. **(L)** Frequency of CD11c^+^MHCII^hi^ cells within the indicated populations. Data are from one experiment representing 4 similar experiments. Data in (J) are pooled from two similar experiments in which qPCR was performed, n = 6 mice per condition.

Interferon-γ is the predominant effector cytokine associated with the activation of phagocytic cells to kill intra-phagosomal pathogens [12]. Treatment of LmLm mice with anti-IFN-γ Ab negated the control of parasite numbers on day 4 post-ch. (Fig 6A) and significantly reduced the frequency of CD11b^+^ per ear (Fig 6B), although this did not reach significance when the absolute number of cells was compared (Fig 6C). Importantly, treatment significantly reduced both the frequency (Fig 6D) and absolute number (Fig 6E) of Ly6C^+^CCR2^+^CX_3_CR1^+/-^ inflammatory or Ly6C^+^CCR2^-^CX_3_CR1^+^ inflammatory monocyte derived cells in LmLm mice, demonstrating that IFN-γ drives a proportion of monocyte recruitment during secondary infection. Treatment of LmLm mice dramatically decreased the frequency of CD11b^+^iNOS^+^ cells to levels observed in naïve mice (Fig 6F) and reduced the absolute number of CD11b^+^iNOS^+^ cells 14-fold (Fig 6G) and the number of iNOS^+^ inflammatory monocytes 20-fold (Fig 6H). Treatment also dramatically reduced the proportion of monocytes in the skin that were iNOS^+^ (Fig 6I).

Anti-IFN-γ Ab did not alter *ifn-*γ gene expression but did reduce the production of *inos*, *cxcl10*, and *cxcl9* (Fig 6J), suggesting that CXCL10 production induced by IFN-γ is driving monocyte recruitment. Blockade of IFN-γ in LmLm mice resulted in MHCII down regulation on RFP^+^ monocytes to levels that were indistinguishable from NaLm mice and significantly lower than LmLm mice treated with control Ab (Fig 6K). Similar to NaLm mice, RFP^+^ monocytes from anti-IFN-γ Ab treated LmLm mice also had lower percentages of CD11c^+^MHCII^hi^ expressing cells versus RFP^-^ monocytes from the same site of infection, while control LmLm mice had increased frequencies (Fig 6L).

Continuous blockade of IFN-γ in LmLm mice resulted in a prolonged reduction in the frequency of iNOS^+^CD11b^+^ cells and enhanced parasite loads on day 7 p.i. (S6A and S6B Fig). Under these conditions, immune mediated swelling significantly increased (S6C Fig) and neutrophils became both more frequent (S6D Fig, left side) and the predominant infected cell type (S6E Fig). Increased neutrophil-mediated immuno-pathology under conditions of reduced monocyte recruitment has been reported previously following *Toxoplasma gondii* infection in the gut [6]. These observations demonstrate that IFN-γ drives a proportion of the recruitment of Ly6C^+^CCR2^+^ monocytes to the skin and the expression of MHC II and iNOS by these cells.

### CCR2^+^ monocytes are the effector cell responsible for parasite elimination at sites of secondary challenge

**Fig 7:**
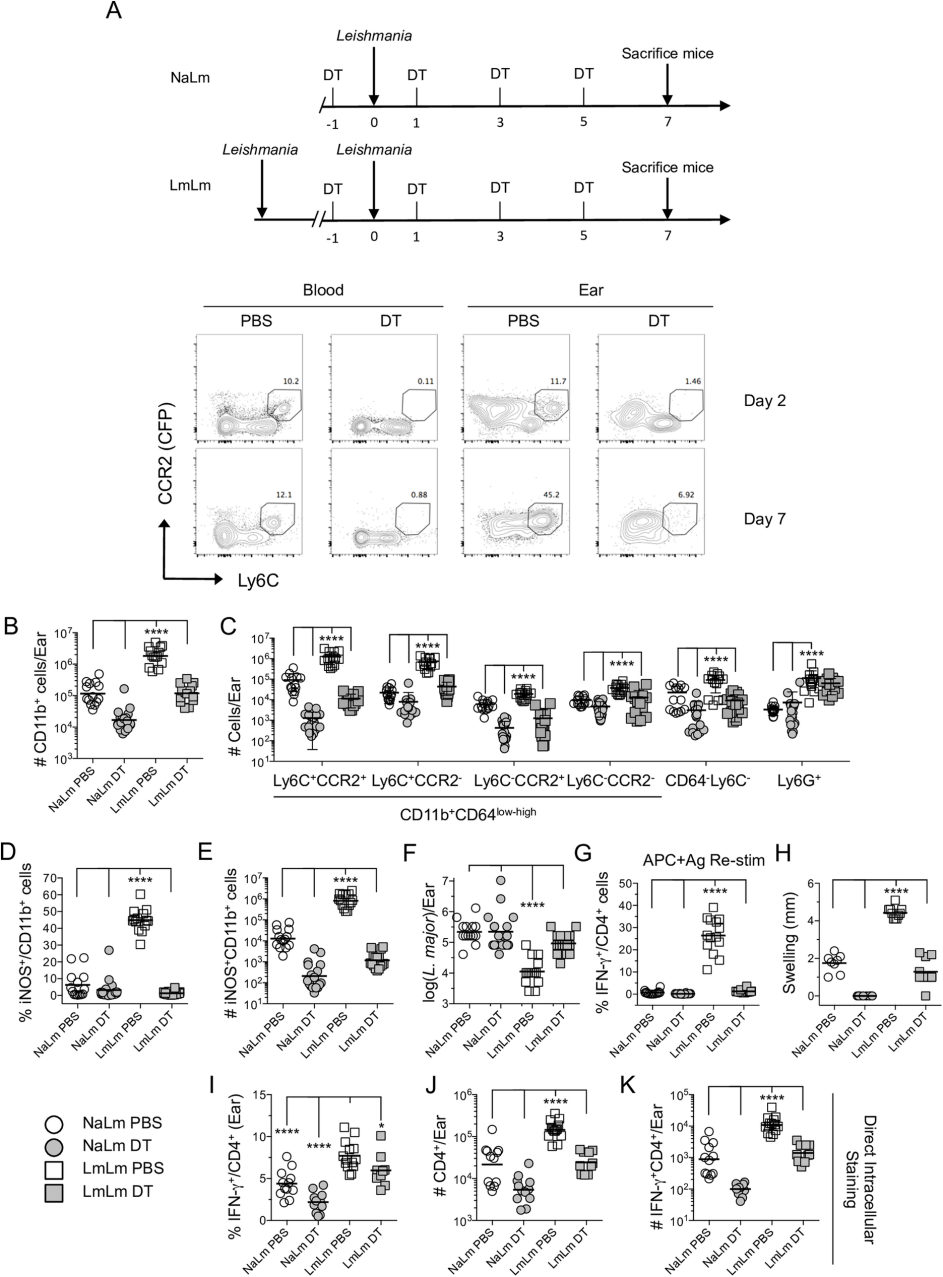
CCR2^+^ Monocytes are the effector cell responsible for parasite elimination at sites of secondary challenge. C57BL/6 CCR2.CFP-DTR ‘depleter’ mice that were uninfected or had a healed primary infection were treated with PBS or DT prior to and every other day following *L*. *major* challenge. **(A)** Schematic of DT treatment and representative CCR2.CFP versus Ly6C plots of CD11b^+^ cells from the blood or ears of naïve mice challenged with *L*. *major* and treated with PBS or DT. **(B and C)** Total absolute number of CD11b^+^ cells **(B)** or of the indicated population **(C)** per ear on day 7 post-ch. **(D and E)** Percentage **(D)** and absolute total number **(E)** of iNOS^+^ cells within the total CD11b^+^ population. **(F)** Number of parasites per ear as determined by limiting dilution analysis. **(G)** Percentage of IFN-γ^+^ cells within the TCRβ^+^CD4^+^ population following APC+Ag stimulation of ear-derived cells. Data are pooled from two repeat experiments employing a total of 14–16 ears per group. **(H)** Ear swelling on day 7 p.i. Data are from one experiment representing two repeat experiments. **(I-K)** Direct intracellular staining for the frequency of IFN-γ^+^ cells per ear **(I)** the absolute number of TcRβ^+^CD4^+^ per ear **(J)**; or the absolute number of IFN-γ^+^ TcRβ^+^CD4^+^ cells per ear **(K)**. Bar represents the Mean **(D, G, H, and I)** or Geometric mean **(B, C, E, F, J and K)**. Data are pooled from two repeat experiments. Level of significance is indicated in the materials and methods.

We next employed CCR2-diptheria toxin receptor (DTR) mice in which CCR2 expressing cells also express the DT receptor to formally demonstrate the role of CCR2^+^ cells at secondary sites of infection (Fig 7A). Analysis of the skin and blood revealed short-term DT treatment efficiently eliminated CCR2 expressing cells (Fig 7A). Following DT treatment we observed highly significant decreases in the total number of CD11b^+^ cells per ear in both NaLm and LmLm mice 7 days post-challenge (Fig 7B). These decreases were largely attributed to the absence of Ly6C^+^CCR2^+^ inflammatory monocytes, and Ly6C^+^CCR2^-^ inflammatory monocyte-derived cells, but also extended to other cell types (Fig 7C), confirming that during the inflammatory response at a dermal site of infection, CCR2^+^ monocytes undergo maturation and populate these cellular niches. As expected, the depletion of CCR2^+^ cells in DT treated mice resulted in a corresponding increase in the frequency of other CD11b^+^ cell types in the skin versus non-depleted mice, most notably neutrophils (S7A Fig). Depletion of CCR2^+^ cells also led to a dramatic decrease in the frequency and total number of iNOS^+^ cells in the ear of LmLm mice (Fig 7D and 7E) and a loss of parasite killing (Fig 7F). The decrease in the number of iNOS^+^ cells was most dramatic in the Ly6C^+^CCR2^+^ and Ly6C^+^CCR2^-^ populations (S7B Fig, note log axis), confirming that CCR2^+^ inflammatory monocytes or inflammatory monocyte derived cells are largely responsible for iNOS production and parasite killing at a site of secondary infection.

Remarkably, DT treatment also led to a dramatic decrease in the frequency of skin-derived CD4^+^ T cells with the capacity to produce IFN-γ upon *ex-vivo* antigen-restimulation (Fig 7G), an observation that was reflected by the absence of immune mediated swelling at the challenge site (Fig 7H). To investigate this further we employed direct intracellular staining (dICS) [20] and counting beads to enumerate the total number and number of *in-situ* IFN-γ producing CD4^+^ T cells per ear (Fig 7I–7K). We found a small but significant reduction in the frequency of *in-situ* IFN-γ^+^ cells at the challenge site of DT- versus PBS-treated LmLm animals; suggesting CCR2-derived cells play a role in antigen presentation in the skin (Fig 7I). In addition, we observed a large and significant reduction in both the total number of CD4^+^ T cells per ear (Fig 7J) and the total number of CD4^+^IFN-γ^+^ cells per ear (Fig 7K) in LmLm DT-treated mice. This reduction was not due to off-target depletion of T cells in CCR2.CFP-DTR mice following DT treatment as the number of CD4^+^ T cells in the dLN was the same in PBS versus DT treated groups (S8A Fig). In addition, DT treatment of wild-type litter-mate controls did not alter the total number, or number of IFN-γ^+^ T cells in the ear versus PBS treated CCR2.CFP-DTR mice (S8B and S8C Fig), suggesting DT treatment did not alter the CD4 T cells response. Therefore, in addition to parasite phagocytosis and killing at a secondary site of challenge, monocytes are also important for down stream elicitation of adaptive immunity, as previously shown [10].

While we were initially surprised that parasites were able to maintain infection in the absence of monocytes acting as host cells following DT treatment, this is likely due to the compensatory infection of neutrophils in these animals, which appear to provide a safe haven for *Leishmania* (S7C Fig), similar to our observation following anti-IFN-γ treatment (S6E Fig). Therefore Ly6C^+^CCR2^+^ inflammatory monocytes are the primary cell involved in parasite killing at secondary sites of challenge.

## Discussion

Following infection with intra-phagosomal pathogens, inflammatory cell recruitment may favour the establishment of disease due to the provision of permissive host cells [12,16]. In the context of *Leishmania* infection, where the majority of parasites initially infect neutrophils, immature inflammatory monocytes would appear to be an ideal secondary target cell versus pre-existing mature tissue macrophages and dendritic cells. We were able to define CD11b^+^CD24^low^CD64^+^Ly6C^+^CCR2^+^CX_3_CR1^+^ inflammatory monocytes as the preferred mononuclear host cell for *L*. *major* following the initial neutrophil phase of infection. This contrasts our previous data that relied largely on Ly6C expression and reported only 10–20% of infected cells were inflammatory monocytes at early time points [17], emphasizing the need to use additional markers to track these cells. Following infection, parasite proliferation was delayed until the monocyte phase of infection, and sorted monocytes supported replication *ex-vivo*. Similar results were observed employing *in-vitro* infection of human monocytes. CD11b^+^CD64^low-high^Ly6C^-^CCR2^-^Ly6G^-^ macrophages or CD11b^+^CD64^-^Ly6C^-^ DCs that, based upon their phenotype and presence in naïve ears prior to challenge are pre-existing resident cells, accounted for a remarkably small proportion of infected cells at early time points p.i. CX_3_CR1^+^Ly6C^-^ ‘patrolling’ monocytes were also rarely infected.

Inflammatory monocyte infection by *L*. *major* at day 3 post primary infection was associated with lower expression levels of MHCII versus uninfected cells. Because inflammatory monocytes originate as MHCII negative cells in the blood, this strongly suggests that infection prevents their maturation to MHCII^+^ cells, contrasting the more conventional macrophage-centric model in which infection down-regulates MHCII^+^ expression in already mature cells. A potential implication of these observations is that *in-vitro* infection of monocytes, rather than matured macrophages, may be a more relevant model with which to study phagocyte modulation by the *Leishmania* parasite.

A common property of intra-phagosomal pathogens is their relatively slow rate of replication and dependence on the priming of CD4^+^ ‘Th1’ cell-mediated adaptive immunity for host resistance [12]. Despite considerable knowledge around the requirements for efficient Th1 CD4 T cell priming and memory cell generation, effective vaccination against any intra-phagosomal pathogen has been elusive [29–32]. The results presented here suggest a rapid response is required to counteract the modulation of immature inflammatory monocytes by *Leishmania*. At sites of secondary infection, iNOS^+^ cells were exclusively inflammatory monocytes or very recently inflammatory monocyte derived cells, not tissue resident cells. While iNOS production, increased MHCII expression, and maturation to a DC phenotype was IFN-γ dependent, the vast majority of iNOS^+^ cells maintained a largely immature phenotype, contrasting with observations in the dLNs of chronic sites of primary infection where iNOS production is associated with a CD11c^+^MHCII^hi^ mature DC phenotype [5]. Therefore, monocytes can acquire functionality without differentiation to a macrophage or DC phenotype *in-vivo*, similar to observations describing their role in steady state antigen transport [1].

Infected monocytes purified from dermal sites of secondary infection contained intact parasites that failed to expand upon *in-vitro* culture. These observations are in agreement with those of Muller et. al. [33], in which iNOS production was shown to suppress parasite metabolism without necessarily resulting in direct parasite killing. In addition, while we found significant correlation between infection and iNOS production within monocytes during secondary infection, we also found high frequencies of iNOS^+^ monocytes within the RFP^-^ population; supporting the idea that IFN-γ can also induce iNOS production by uninfected bystander cells [34].

Our results mirror those reported by Reiner et al. [35] in which pre-exposure of human monocytes to IFN-γ prevented *Leishmania*-induced inhibition of monocyte activation and extend the idea that CD4 T cell-mediated immunity is a major factor in shaping innate inflammation [36,37]. Our observations also bear similarity to studies examining the role of vaccine-induced immunity in shaping the innate inflammatory response in the spleen and liver or stomach, specifically the activation of inflammatory monocytes as a strong correlate of protective immunity [38,39].

CCR2-depleter mice allowed for the timed removal of monocytes following primary infection but prior to secondary challenge. Our results demonstrate that blood-derived CCR2^+^ monocytes are the definitive source of iNOS^+^ cells and are essential for rapid parasite elimination at a secondary site in infection. They also revealed the extensive degree to which these cells undergo maturation and populate numerous phenotypic niches [3]. Interestingly, we saw no evidence for parasite killing by neutrophils in the absence of monocytes and repeatedly found that neutrophils were acting as a safe haven for parasites, similar to their role in primary *L*. *major* infection.

Our observations extend a model of disease whereby *Leishmania* parasitizes the innate inflammatory response in the skin following infection [15,40]. The need for a rapid immune response to condition monocytes immediately upon infection may explain the failure of conventional vaccination strategies that generate memory cells against intra-phagosomal pathogens. In these settings, the time required for the expansion and acquisition of effector function by memory cells residing in secondary lymphoid organs may be too long to counteract pathogen-mediated monocyte modulation.

## Materials and methods

### Parasites and parasite quantification

Generation, maintenance and metacyclic promastigote purification of *L*. *major* (MHOM/IL/80/Friedlin) or *L*. *major*-RFP was performed as described previously [15]. Parasite loads were determined by limiting dilution analysis (LDA). Briefly, two fold serial dilutions in 96-well flat bottom microtiter plates were performed by overlaying 100μl of the diluted tissue suspension onto 50 μl NNN medium containing 20% defibrinated rabbit blood. The dilutions were made in duplicate. The plates were scored microscopically for growth and the number of parasites in each tissue was determined from the highest dilution at which parasites could be grown out after 7–10 days incubation at 26°C. For two-fold limiting dilution of known numbers of sorted RFP^+^ infected cells, cells were plated in a multiple of two (typically 256 cells in the first well) in quadruplicate based on the number of available cells post-sorting.

### Mice

Female C57BL/6 WT and B6.129P(Cg)-*Ptprc*^a^ *Cx3cr1*^tm1Litt^/LittJ (Jax strain 005582, CX3CR1-GFP) were originally obtained from Jackson Laboratories. CX_3_CR1-GFP mice were kindly provided by Drs. John Grainger and Yasmine Belkaid (Laboratory of Parasitic Diseases, NIAID, Bethesda, MD) or Dr. Paul Kubes (Snyder Institute for Chronic Diseases, University of Calgary, Calgary, AB). *cx3cr1*^gfp/+^ mice (B6.SJL-Cd45a(Ly5a)/Nai x B6.129P(Cg)-*Ptprc*^a^ *Cx3cr1*^tm1Litt^/LittJ F1) were bred in house. C57BL/6 LYS-eGFP knock-in mice (TAC 0342) were obtained through a supply contract between the National Institute of Allergy and Infectious Diseases (NIAID) and Taconic Farms. CCR2-depleter mice (CCR2.CFP.DTR mice) were kindly provided by Dr. Eric G. Pamer (Memorial Sloan Kettering Institute, New York, NY). All mice were bred and maintained at the University of Calgary Animal Resource Centre or at the NIAID animal care facility under specific pathogen-free conditions.

### Primary infection and challenge

Chronically infected mice were generated by injecting 10^4^ *L*. *major* metacyclic promastigotes subcutaneously in the hind footpad in a volume of 40μl and used at 12 to 20 weeks post-primary infection when footpad lesions had completely resolved. Naïve mice and mice with a chronic primary infection, were challenged with 2x10^5^ *L*. *major-*RFP metacyclic promastigotes intra-dermally (i.d) in the ear in a volume of 10μl.

### Sand fly infection

Two-to-four day old *Phlebotomus duboscqi* females were obtained from a colony initiated from field specimens collected in Mali. Flies were infected by artificial feeding through a chick skin membrane on heparinized mouse blood (drawn intracardially from BALB/c mice) containing 5.10^6^ *L*. *major* promastigotes/ml of blood. Blood engorged flies were separated and maintained at 26°C and 75% humidity and were provided 30% sucrose ad libitum. After 13–14 days, 9–10 flies per experimental group were anesthetized with CO_2_, killed in 5% soap solution, and whole midguts, including the crop, were dissected and transferred into tubes containing 25 μl 1× PBS. The guts were macerated briefly using a plastic pestle. A 10-μl sample of the supernatant was counted under a hemocytometer and the numbers of metacyclic promastigotes, non-metacyclic forms, and total parasite number, as determined by morphology and movement, were counted.

### Exposure of mice to infected or uninfected sand flies

*Leishmania* infections were allowed to mature for 14–16 days within the sand fly midgut. One day before transmission the sucrose diet was removed. On the day of transmission, 4–5 flies were transferred to small plastic vials (volume 12.2 cm2, height 4.8 cm, diameter 1.8 cm) covered at one end with a 0.25-mm nylon mesh. Mice were anesthetized by intraperitoneal injection of 30μl of ketamine/xylazine (100 mg/ml). Specially designed clamps were used to bring the mesh end of each vial flat against the ear, allowing flies to feed on exposed skin for a period of 2–3 hours in the dark at 23°C and 50% humidity.

### Tissue processing and monocyte purification

Ear tissue was prepared as previously described [13,20]. In experiments employing direct intracellular staining (dICS), ears were removed and placed in 70% ethanol for 2–5 minutes and then allowed to dry. Separated dorsal and ventral sheets of ears were then incubated at 37°C for 90 minutes in 1ml of DMEM containing 20μg/ml of Brefeldin A and 16μg/ml of Liberase that was pre-warmed to 37°C. Following Liberase treatment tissue was homogenized for 3½ minutes in a Medicon using a Medimachine (Becton Dickinson). The tissue homogenate was then flushed from the medicon with 10 ml RPMI media containing 0.05% DNase I and filtered using a 50 μm-pore-size cell strainer. In experiments employing dICS, 2μg/ml of Brefeldin A was added to DNase media pre-warmed to 37°C and the ear homogenate returned to 37°C and 5% CO_2_ for an additional 1.5–2 hours in media containing 2μg/ml of Brefeldine A. Ear draining LN (dLN) were removed and homogenized with a syringe plunger, and the cell suspension was filtered through a 70-μm strainer. Blood was obtained by intra-cardiac bleed and red blood cells were eliminated by using ACK lysing buffer (Lonza).

For monocyte purification, ear tissue was prepared as described above 60h post-infection, and CD11b^+^Ly6C^int/hi^CX3CR1^+^LY6G^-^ infected (RFP^+^) and uninfected (RFP^−^) monocytes were sorted from dermal tissue using a FACSAria (BD Biosciences) cell sorter. Cells were either stained with Giemsa following cytospin directly post-sort to determine intracellular infection or resuspended in complete RPMI and incubated at 37°C for 24h and 48h for subsequent analysis.

### Re-stimulation of tissue-derived cells for cytokine analysis, immunolabelling and flow cytometry

Tissue derived cells were re-stimulated as described previously [22]. Briefly, single-cell suspensions were stimulated with 10^6^ T cell-depleted (Miltenyi Biotech) naïve spleen cells (APCs), with 50 μg/ml freeze-thaw *Leishmania major* antigen. During the final 4–6 hours of culture, 1μg/ml of Brefeldin A (Sigma) was added.

For immunolabelling, cells were washed and labeled with Live/Dead fixable BLUE or AQUA at a 1:500 dilution of the manufacturer suggested stock solution (Invitrogen) to exclude dead cells. Cells were incubated with anti-Fc III/II (CD16/32) receptor Ab (2.4G2), followed by surface staining with various combination of the following antibodies for 30min at 4°C in the dark: PE-Cy7 anti-Ly6G (1A8); -CD4 (RM4-5), APC or BV650 anti-MHCII (M5/114.15.2), APC-eFluor 780 anti-Ly6C (HK1.4), Brilliant Violet 421, APC or PE-Cy7 anti-CD64 (X54-5/7.1), -TCR-β (145-2C11), PerCp-Cy5.5 or Brilliant Violet 605 CD11b (M1/70), Brilliant Violet 785 anti-CD11c (N418), FITC or BV421 anti-CD24 (M1/69), and/or PE-Cy7 anti-CD86 (GL-1). Staining for CCR2 employed AlexaFluor 700 anti-CCR2 (475301) and was done prior to surface staining at 37°C for 20-30min. In some experiments, cells were then fixed with BD Cytofix/Cytoperm (BD Biosciences) according to the manufacturer instructions and stained with FITC or APC anti-IFN-γ (XMG1.2), or Alexa Fluor 488 or APC anti-nos2 (CXNFT). Isotype controls employed were rat IgG1 (R3-34) and rat IgG2b (A95-1 or eBR2a). All Abs were from eBiosciences, BD Biosciences, Biolegends or R&D systems. Data were collected using FACSDiva software on a FACSLSRII or FACSCANTO II flow cytometer (BD Biosciences), and analyzed using FlowJo software (TreeStar). Forward-scatter and side-scatter width was employed to exclude cell doublets from analysis. To determine the absolute number of cells, a portion of each sample was removed for counting with AccuCheck Counting Beads (Invitrogen) as described previously [41].

### Chemicals and reagents

Diphtheria toxin (DT) was obtained from Milipore, reconstituted at 1 mg/ml in PBS, and frozen at −80°C. Mice received 25 ng/g DT via the intra-peritoneal route in 0.2–0.3 ml PBS. The toxin was injected every other day starting one day prior to the challenge of the mice with *L*. *major* parasites. The efficiency of the depletion was verified 8 days post-depletion, in ears cells, bone marrow and blood.

Anti-IFN-γ monoclonal antibody anti-mouse clone XMG1.2 (BioXCell) was employed to block IFN-γ *in vivo*. A single dose of 0.5 mg/mL of XMG1.2 was administered by intraperitoneal injection 2h before the infection. The mice euthanized 7 days post-infection were also treated with the same dose of the mAb 4 days after infection. As an isotype control we treated the mice with 0.5 mg/mL of a mAb rat IgG1 anti Horseradish Peroxidase (BioXCell).

### Blood donors and human monocyte isolation and in-vitro infection

Human peripheral blood monocytes were obtained from healthy volunteers by counterflow centrifugal elutriation at the NIH Blood Bank under Institutional Review Board–approved protocols of NIAID and the Department of Transfusion Medicine. Human monocytes *in vitro* culture were performed in RPMI 1640 medium (Life Technologies) supplemented with 10% heat-inactivated FCS, 4 mM L-glutamine, 10 mM HEPES, 100 U/ml penicillin and 100 μg/ml streptomycin. Experiments were carried out on the day of collection. Monocytes were plated at 10^5^/ml cells in 6-well tissue culture plates and infected with *L*. *major* metacyclic promastigotes at a multiplicity of infection (MOI) of 1:4. At 6 hours, excess parasites were removed by two washes at cell speed. At 6 and 72h cells were recovered and cytospin and stained with Diff quick. ROS production was measured as previously described [42].

### Two-photon intravital imaging and image analysis

Sequential two-photon intravital imaging of single bite sites and image analysis was performed as reported and described previously [22,43]. Briefly, anesthetized mice were imaged in the lateral recumbent position that allowed the ventral side of the ear pinna to rest on a coverslip. A strip of Durapore tape (3 M) was stuck to a bench top several times (to ensure that subsequent removal would not cause undue damage) and placed lightly over the ear pinna and affixed to the imaging platform in order to immobilize the tissue. Care was taken to minimize pressure on the ear.

Images were acquired using an inverted LSM 510 NLO multiphoton microscope (Carl Zeiss Microimaging) enclosed in an environmental chamber that was maintained at 30°C. This system had been custom fitted with 3 external non-descanned PMT detectors in the reflected light path. Images were acquired using either a 20×/0.8 air objective or a 25×/0.8 NA water immersion objective. Fluorescence excitation was provided by a Chamelon XR Ti:Sapphire laser (Coherent) tuned to 920 nm for eGFP and RFP excitation. Voxel dimensions were 0.64×0.64×2 μm using the 20× objective and 0.36–0.51×0.36–0.51×2 μm using the 25× objective.

Raw imaging data were processed with Imaris (Biplane) using a Gaussian filter for noise reduction. All images are displayed as 2D maximum intensity projections.

### Real-time PCR

For analysis of cytokine and chemokine gene expression ears cells were stored in RLT buffer at -80°C until the day of the RNA extraction. Homogenates were then passed through QIAshredder columns, and RNA was purified using an RNeasy midikit according to the manufacturer’s protocol (Qiagen). Reverse transcription was performed using the SuperScript III first-strand synthesis system for reverse transcription-PCR (RT-PCR) (Invitrogen Life Technologies). Real-time PCR was performed on an ABI Prism 7900 sequence detection system using the primer probe sets designed by Applied Biosystems. The quantity of the product was determined by the comparative threshold cycle method using 2^−ΔΔCT^ (where CT represents the cycle threshold) to determine the fold increase. Each gene was normalized to the 18S rRNA endogenous control and to the average ΔCT of naive mice as sample control.

### Statistics

Data were compared using the student’s t-test. Comparisons between multiple groups were done using ANOVA with Holm-Sidek’s post-test. Parasite load determined by LDA and absolute number data were log transformed before statistical analysis. All p-values are two-sided. Statistical calculations were done in Graphpad PRISM 6.0 (www.graphpad.com). **** p<0.0001; *** 0.0001< p<0.001; ** 0.001<p<0.01; * 0.01<p<0.05.

### Ethics statement

Human peripheral blood monocytes were obtained from healthy volunteers at the NIH Blood Bank under Institutional Review Board–approved protocols of NIAID and the Department of Transfusion Medicine. All experiments were approved by the University of Calgary Animal Care Committee (Protocol number AC14-0142) in compliance with the Canadian Council for Animal Care or by the NIAID animal care and use committee (Protocol no. LPD68E) in compliance with the Animal Welfare Act and the PHS Policy on Humane Care and Use of Laboratory Animals.

## Supporting information

S1 Fig Flow cytometry gating strategy for data depicted in Fig 1A–1C. Ear derived cells were stained with the indicated antibodies for analysis by flow cytometry. **(A)** Cells were gated based on forward and side scatter, doublets were excluded employing FSC-W and SSC-W, dead cells were excluded employing a LIVE/DEAD dye, and gated on CD11b^+^ cells. **(B)** MHCII, CD11c, and CCR2 expression on the indicated populations identified employing Ly6C and CX_3_CR1. **(C)** Representative RFP expression by CD11b^+^ cells derived from ears inoculated with *L*. *major*-RFP or *L*. *major*-WT parasites.
(TIF)

S2 Fig Sand fly bites elicit recruitment of neutrophils and inflammatory monocytes to the skin. **(A)** Representative flow cytometry contour plots of Ly6G and Ly6C expression on Live CD11b^+^ cells (gated as in S1A Fig) from the ear skin 18 hours post-exposure to the bites of uninfected *P*. *duboscqi* sand flies. **(B)** Mean frequency of CD11^+^Ly6G^+^Ly6C^int^ neutrophils, Ly6G^-^Ly6C^hi^ inflammatory monocytes, or Ly6G^-^Ly6C^negative/low^ macrophages/DCs as indicated in (A) as a proportion of total CD11b^+^ cells. **(C)** Mean relative number of the indicated populations per ear. **(D)** Mean frequency of CD11c^+^MHCII^hi^ dendritic cells (DCs) within the indicated populations. n = 7 (naïve) or 8 (sand fly exposed) total ears. Data is pooled from 2 independent experiments with similar results. In (B-D) asterisk refers to significant differences between naïve ears and ears exposed to the bites of sand flies.
(TIF)

S3 Fig Flow cytometry gating strategy and analysis of CD11b^+^ cells from naïve mice. Cells were stained with the indicated antibodies for analysis by flow cytometry. **(A)** Representative contour plots of CCR2, Ly6C, and CX_3_CR1 expression on Live CD11b^+^ cells from the blood or ears of naïve C57BL/6 CX_3_CR1-gfp mice. **(B)** Representative MHCII and CD11c expression on CD11b^+^Ly6G^-^Ly6C^+^CCR2^+^ inflammatory monocytes from the blood of naïve mice. **(C)** Gating strategy to define the indicated RFP^+^CD11b^+^ populations following infection with *L*. *major*.
(TIF)

S4 Fig Characterization of human elutriated monocytes employed for infection. Peripheral blood monocytes were obtained from healthy volunteers by counterflow centrifugal elutriation and stained for CD16 and CD14.
(TIF)

S5 Fig Analysis of blood-derived monocytes prior to challenge. Blood was obtained from age-matched naïve mice (NaNa) or mice with a healed but persistent primary infection (LmNa). **(A)** Frequency of CCR2^+^Ly6C^+^CX_3_CR1^+^CD64^low-high^Ly6G^-^ inflammatory monocytes (Infl. Mono.) within the total CD11b^+^ live population. **(B)** Comparison of Ly6C and CCR2 expression levels on CX_3_CR1^+^CD64^low-high^Ly6G^-^CD11b^+^ putative inflammatory monocytes. **(C)** Comparison of MHCII, CD11c, and CD86 expression levels on inflammatory monocytes in the blood from NaNa and LmNa mice. Data is from two pooled experiments employing a total of 15 mice.
(TIF)

S6 Fig Continuous anti-IFN-γ treatment negates iNOS production, blocks parasite killing, and leads to increased frequencies of infected neutrophils on day 7 p.i.. Mice as described in Fig 6 were treated with 0.5mg i.p. anti-IFN-γ or control antibody on day -1 or day -1 and 4.5 and analyzed on day 4 or 7 post-ch. **(A)** Frequency of iNOS^+^ cells within the total CD11b^+^ population. **(B)** Parasite load per ear determined by LDA. In (B) significance refers to LmLm control group versus all other groups. **(C)** Ear swelling at the indicated times post-challenge. Asterisk (*) indicates a significant difference (p≤0.0001) between the LmLm α-IFN-γ group and all other groups from 48h onward. Pound symbol (#) indicates a significant difference (p≤0.017) between the LmLm group and both NaLm groups from 24h onward. **(D and E)** Frequency of the indicated populations within the total CD11b^+^ (D) or CD11b^+^RFP^+^ infected population (E). In (D and E) Asterisk (*) indicates a significant difference (p≤0.002) between the LmLm Control and LmLm α-IFN-γ group.
(TIF)

S7 Fig CCR2^+^ Monocyte deficiency leads to increased proportions of total and infected neutrophils and reduced numbers of iNOS^+^ monocytes and monocyte derived cells. Cells were acquired as described in Fig 7. **(A)** Frequency of the indicated populations within the total CD11b^+^ population. **(B)** Number of iNOS^+^ cells. **(C)** Frequency of the indicated populations within the total RFP^+^ infected CD11b^+^ population. Data is from mice depicted in Fig 7B–7F.
(TIF)

S8 Fig Diphtheria toxin (DT) treatment of CCR2.CFP-DTR or WT litter-mate controls does not alter the CD4^+^ T cell compartment. Cells were acquired as described in Fig 7. **(A)** Total number of CD4^+^ T cells in the dLN of the site of primary (NaLm) or secondary infection (LmLm) in CCR2.CFP-DTR mice treated with PBS or diptheria toxin (DT). Data is from mice depicted in Fig 7I and 7J. **(B)** Total number of CD4^+^ T cells in the dermal site of primary (NaLm) or secondary infection (LmLm) in CCR2.CFP-DTR or wild type (WT) littermate controls (generated from CCR2.CFP-DTR^-/+^ crosses) treated with PBS or DT. Data is from a single experiment employing n = 4–8 ears per group. **(C)** Direct intracellular staining for the absolute number of IFN-γ^+^TcRβ^+^CD4^+^ cells per ear in mice as described in (B).
(TIF)
