# Supplementary figures and images for: Divergent roles for Ly6C+CCR2+CX3CR1+ inflammatory monocytes during primary or secondary infection of the skin with the intra-phagosomal pathogen Leishmania major
Source: PLoS Pathog. 2017 Jun 30;13(6):e1006479. doi: 10.1371/journal.ppat.1006479 (PMC5509374; doi:10.1371/journal.ppat.1006479)

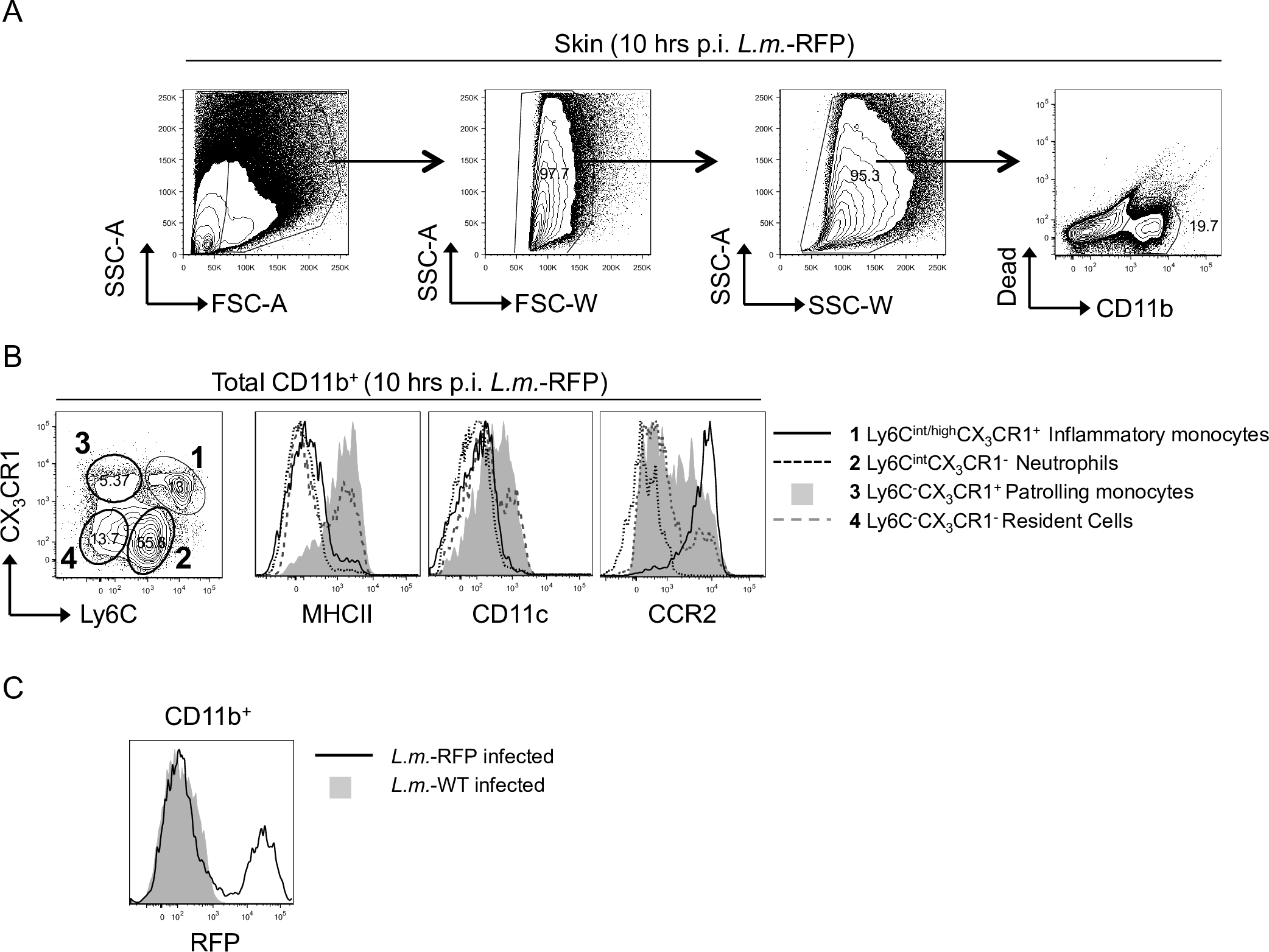

Supplement: S1 Fig — Ear derived cells were stained with the indicated antibodies for analysis by flow cytometry. (A) Cells were gated based on forward and side scatter, doublets were excluded employing FSC-W and SSC-W, dead cells were excluded employing a LIVE/DEAD dye, and gated on CD11b+ cells. (B) MHCII, CD11c, and CCR2 expression on the indicated populations identified employing Ly6C and CX3CR1. (C) Representative RFP expression by CD11b+ cells derived from ears inoculated with L. major-RFP or L. major-WT parasites. (TIF) [file ppat.1006479.s001.tif]

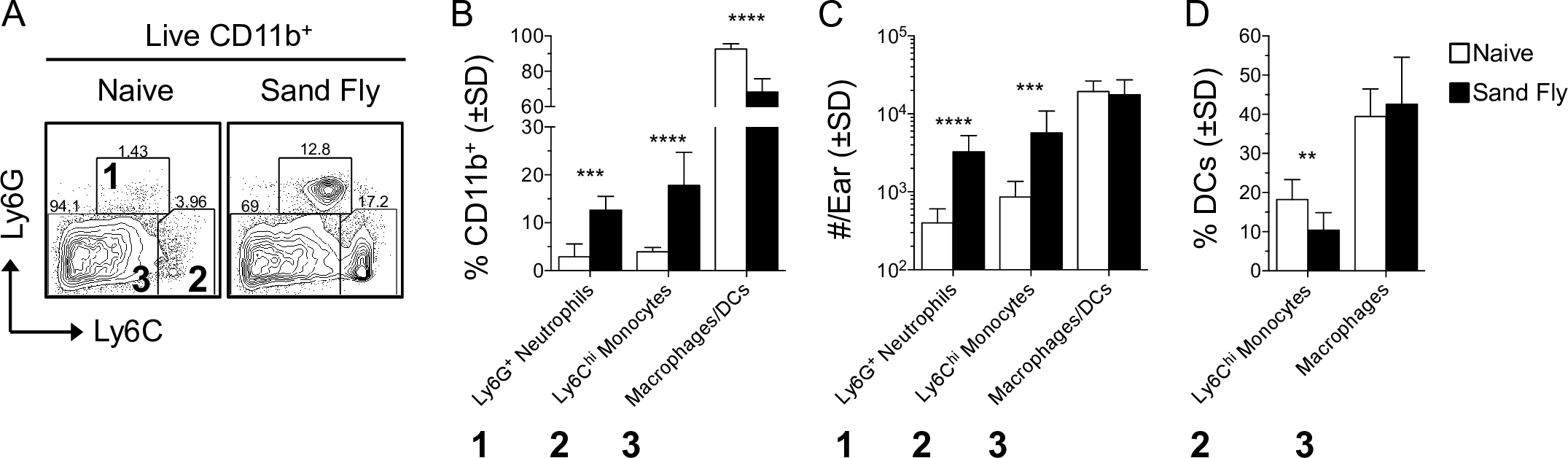

Supplement: S2 Fig — (A) Representative flow cytometry contour plots of Ly6G and Ly6C expression on Live CD11b+ cells (gated as in S1A Fig) from the ear skin 18 hours post-exposure to the bites of uninfected P. duboscqi sand flies. (B) Mean frequency of CD11+Ly6G+Ly6Cint neutrophils, Ly6G-Ly6Chi inflammatory monocytes, or Ly6G-Ly6Cnegative/low macrophages/DCs as indicated in (A) as a proportion of total CD11b+ cells. (C) Mean relative number of the indicated populations per ear. (D) Mean frequency of CD11c+MHCIIhi dendritic cells (DCs) within the indicated populations. n = 7 (naïve) or 8 (sand fly exposed) total ears. Data is pooled from 2 independent experiments with similar results. In (B-D) asterisk refers to significant differences between naïve ears and ears exposed to the bites of sand flies. (TIF) [file ppat.1006479.s002.tif]

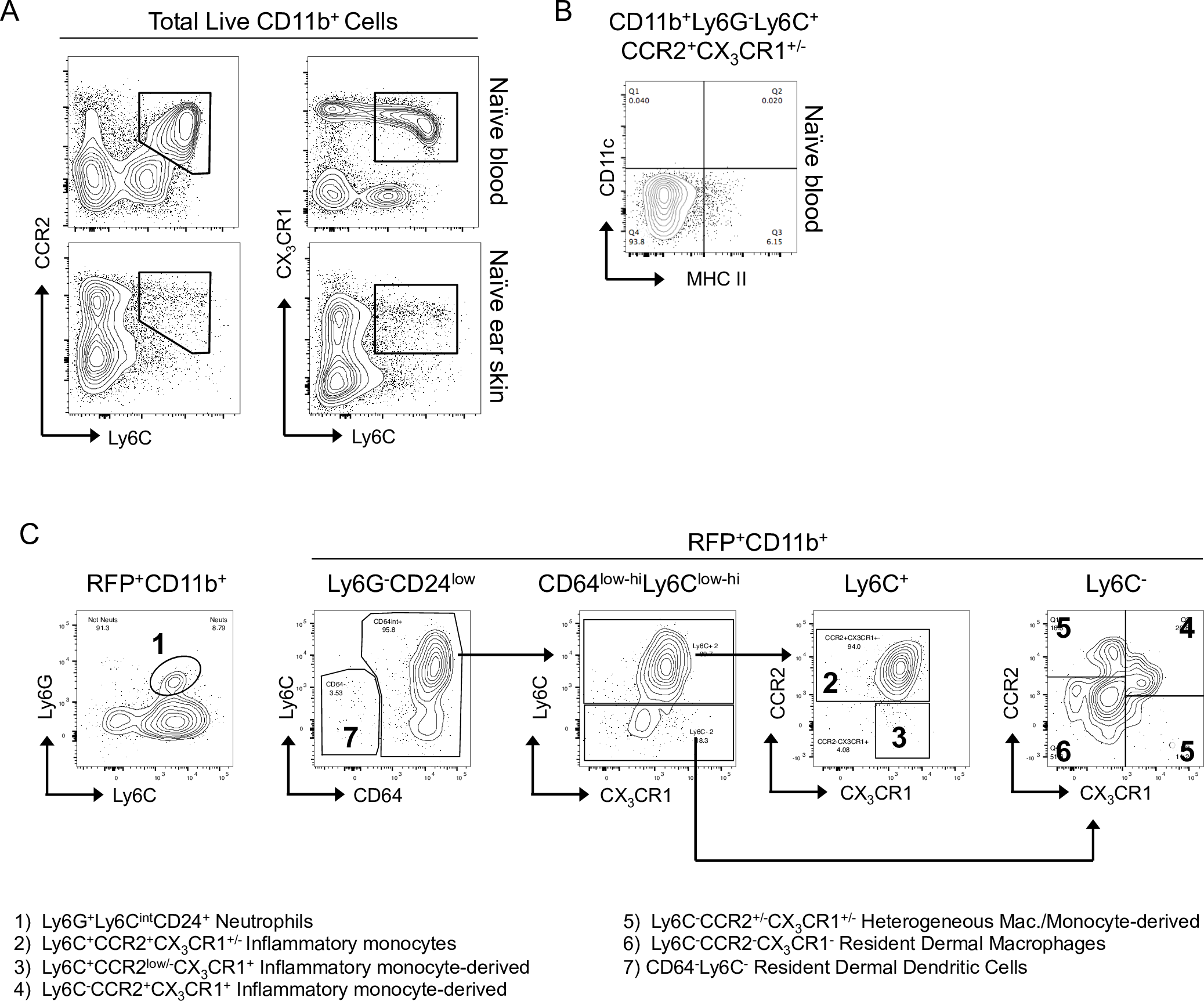

Supplement: S3 Fig — Cells were stained with the indicated antibodies for analysis by flow cytometry. (A) Representative contour plots of CCR2, Ly6C, and CX3CR1 expression on Live CD11b+ cells from the blood or ears of naïve C57BL/6 CX3CR1-gfp mice. (B) Representative MHCII and CD11c expression on CD11b+Ly6G-Ly6C+CCR2+ inflammatory monocytes from the blood of naïve mice. (C) Gating strategy to define the indicated RFP+CD11b+ populations following infection with L. major. (TIF) [file ppat.1006479.s003.tif]

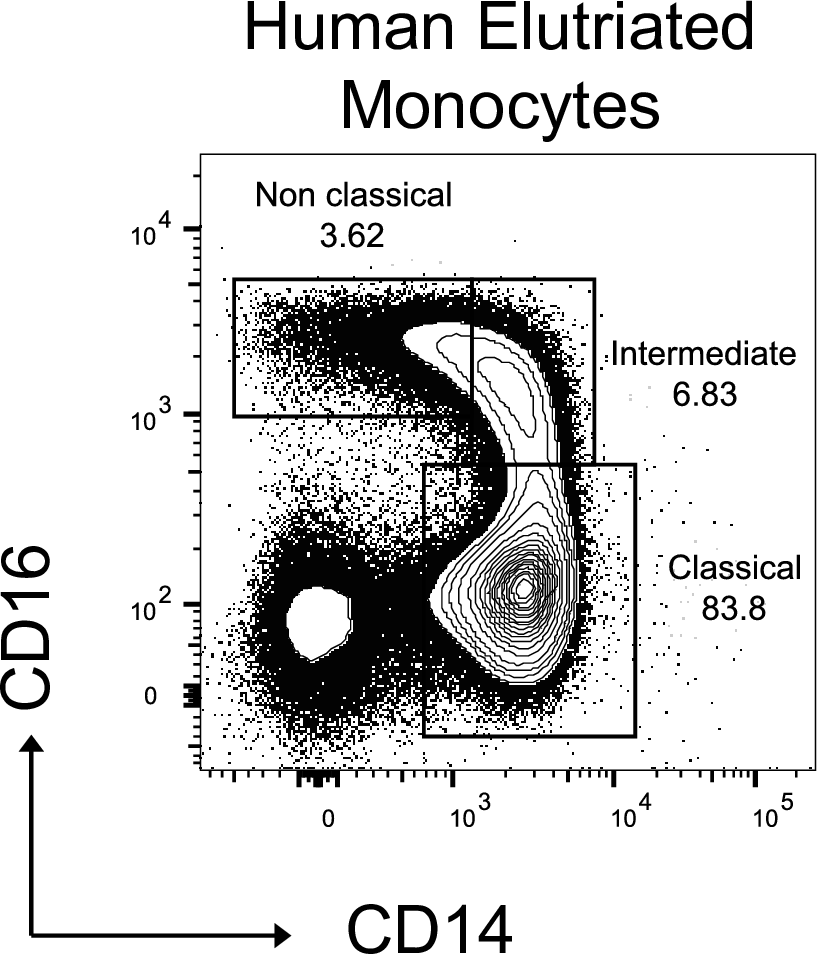

Supplement: S4 Fig — Peripheral blood monocytes were obtained from healthy volunteers by counterflow centrifugal elutriation and stained for CD16 and CD14. (TIF) [file ppat.1006479.s004.tif]

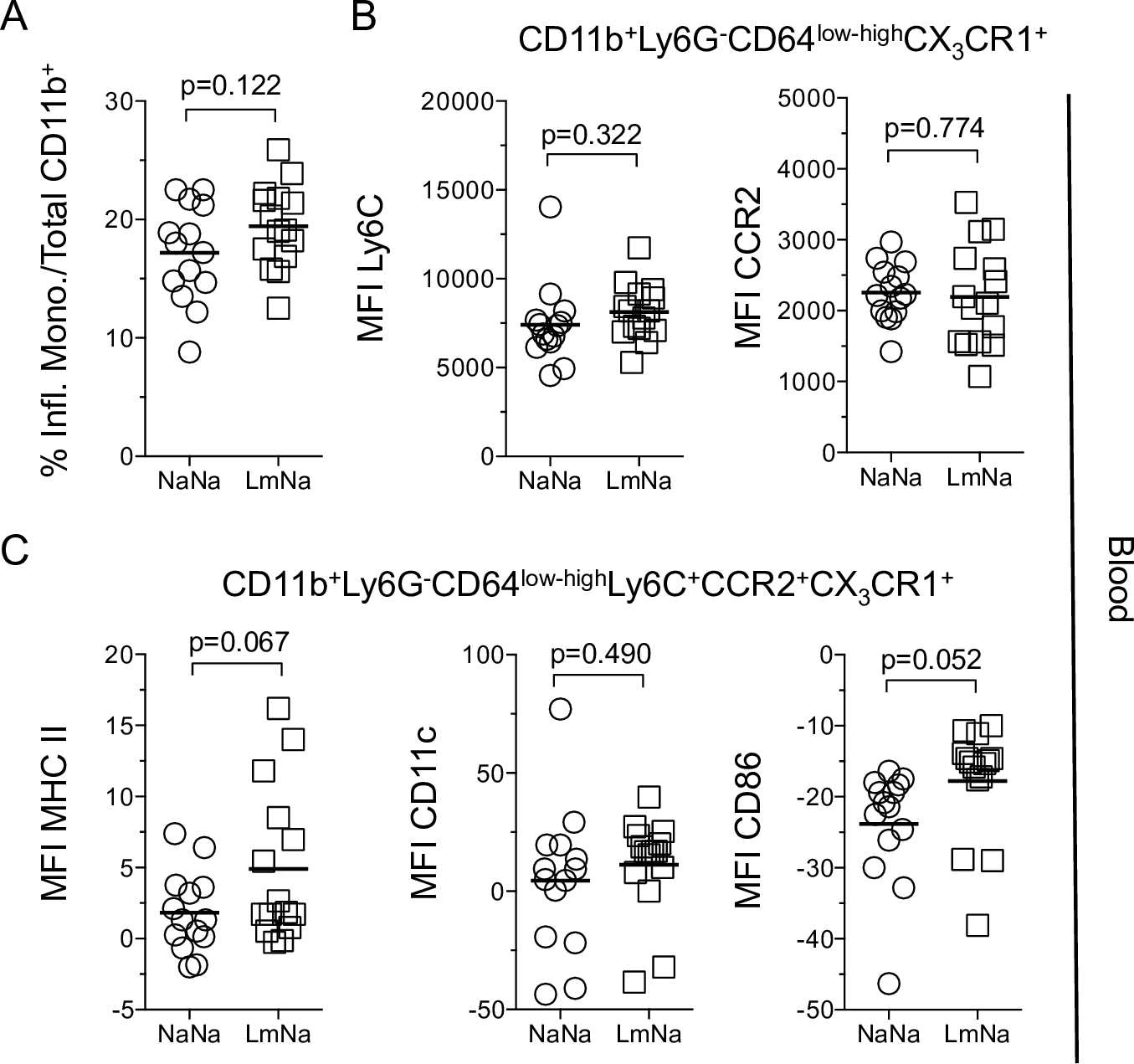

Supplement: S5 Fig — Blood was obtained from age-matched naïve mice (NaNa) or mice with a healed but persistent primary infection (LmNa). (A) Frequency of CCR2+Ly6C+CX3CR1+CD64low-highLy6G- inflammatory monocytes (Infl. Mono.) within the total CD11b+ live population. (B) Comparison of Ly6C and CCR2 expression levels on CX3CR1+CD64low-highLy6G-CD11b+ putative inflammatory monocytes. (C) Comparison of MHCII, CD11c, and CD86 expression levels on inflammatory monocytes in the blood from NaNa and LmNa mice. Data is from two pooled experiments employing a total of 15 mice. (TIF) [file ppat.1006479.s005.tif]

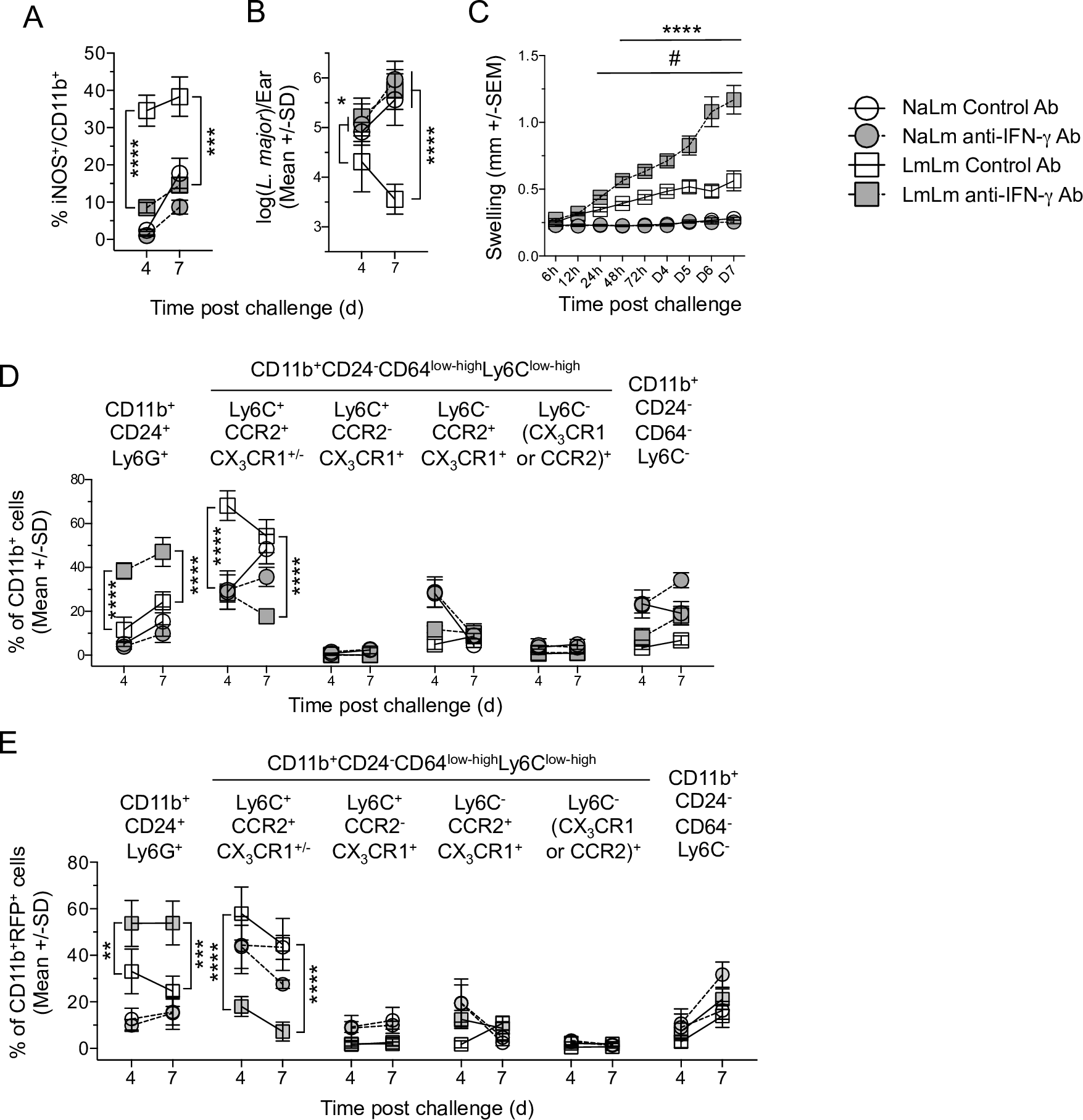

Supplement: S6 Fig — Mice as described in Fig 6 were treated with 0.5mg i.p. anti-IFN-γ or control antibody on day -1 or day -1 and 4.5 and analyzed on day 4 or 7 post-ch. (A) Frequency of iNOS+ cells within the total CD11b+ population. (B) Parasite load per ear determined by LDA. In (B) significance refers to LmLm control group versus all other groups. (C) Ear swelling at the indicated times post-challenge. Asterisk (*) indicates a significant difference (p≤0.0001) between the LmLm α-IFN-γ group and all other groups from 48h onward. Pound symbol (#) indicates a significant difference (p≤0.017) between the LmLm group and both NaLm groups from 24h onward. (D and E) Frequency of the indicated populations within the total CD11b+ (D) or CD11b+RFP+ infected population (E). In (D and E) Asterisk (*) indicates a significant difference (p≤0.002) between the LmLm Control and LmLm α-IFN-γ group. (TIF) [file ppat.1006479.s006.tif]

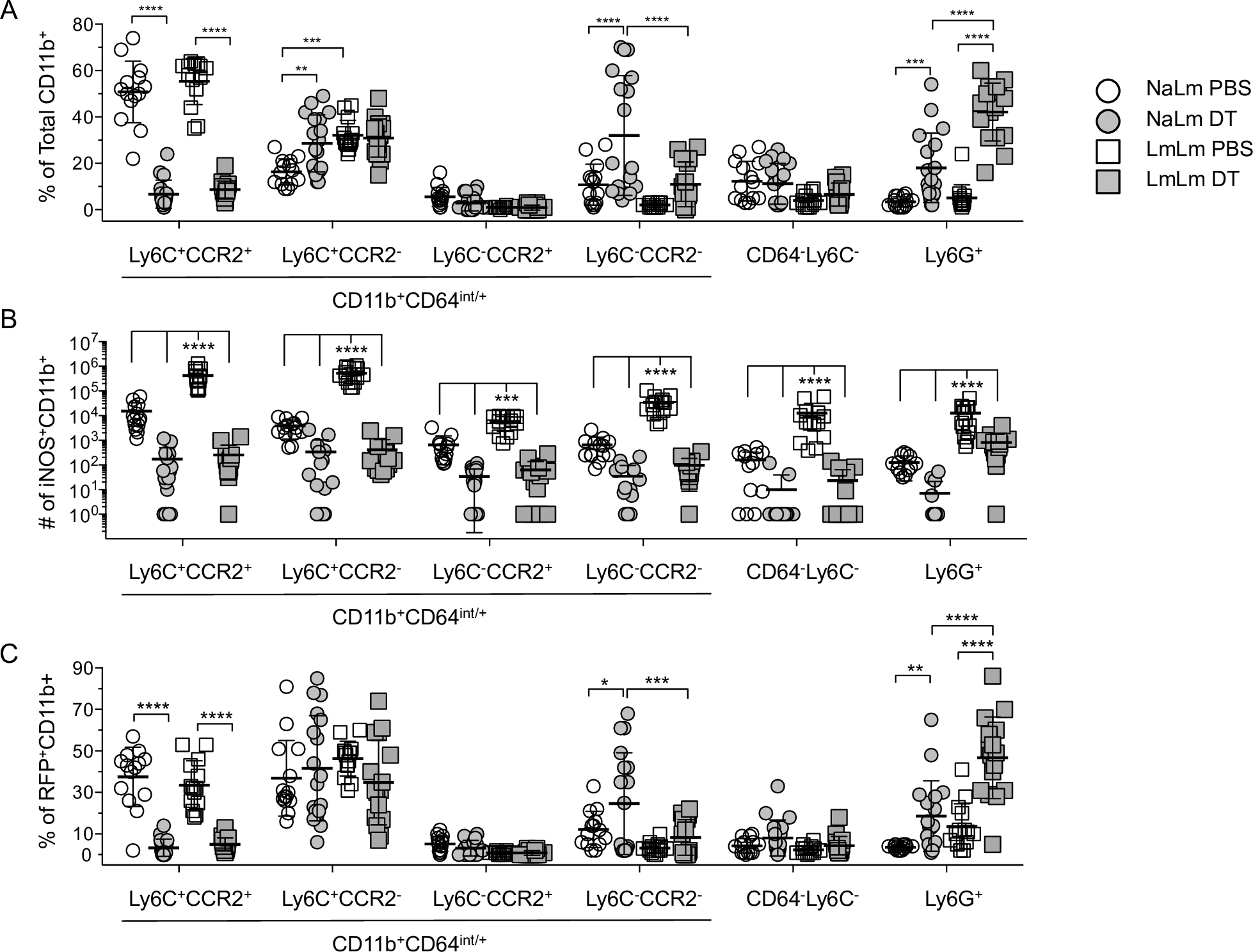

Supplement: S7 Fig — Cells were acquired as described in Fig 7. (A) Frequency of the indicated populations within the total CD11b+ population. (B) Number of iNOS+ cells. (C) Frequency of the indicated populations within the total RFP+ infected CD11b+ population. Data is from mice depicted in Fig 7B–7F. (TIF) [file ppat.1006479.s007.tif]

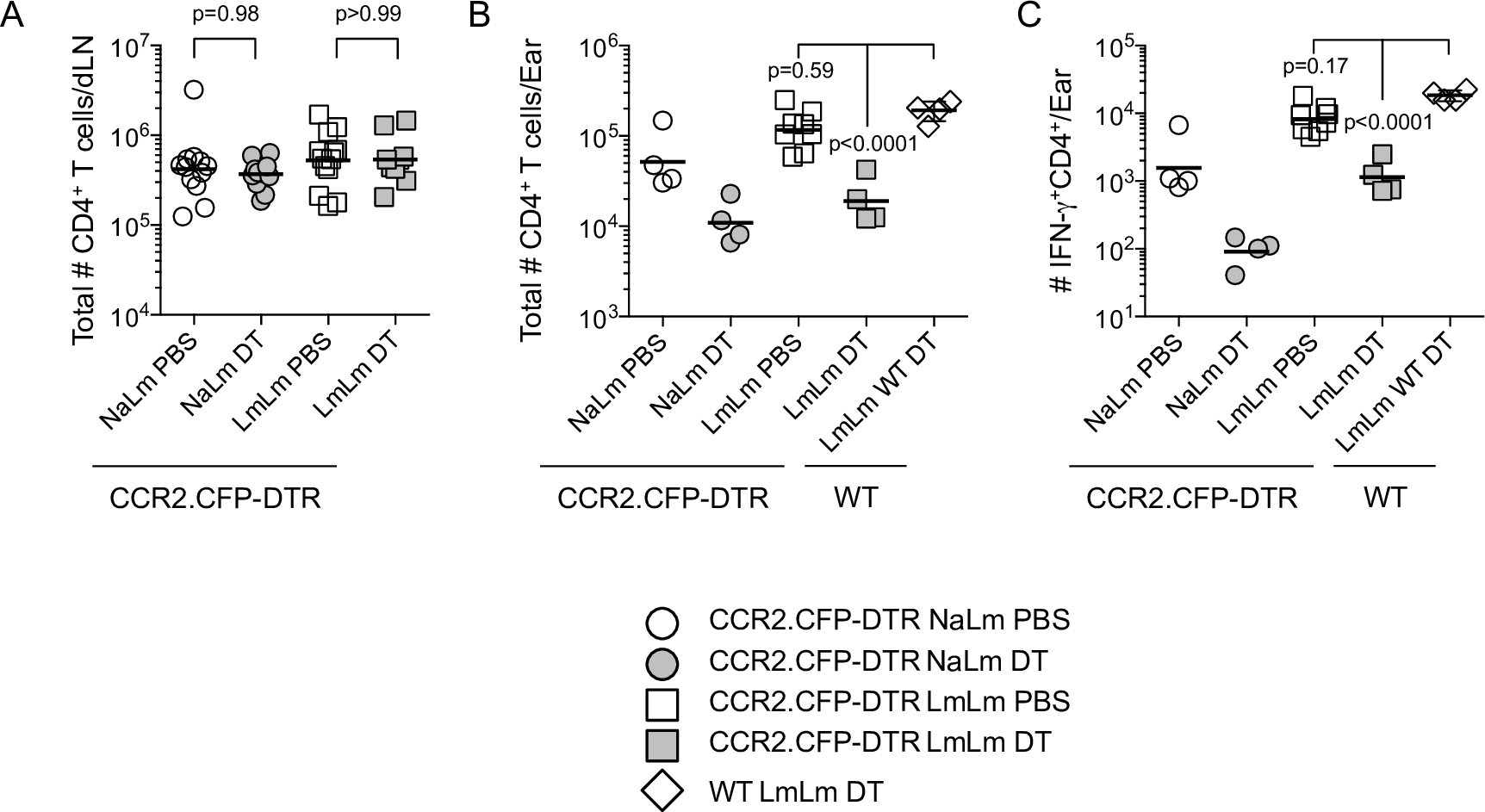

Supplement: S8 Fig — Cells were acquired as described in Fig 7. (A) Total number of CD4+ T cells in the dLN of the site of primary (NaLm) or secondary infection (LmLm) in CCR2.CFP-DTR mice treated with PBS or diptheria toxin (DT). Data is from mice depicted in Fig 7I and 7J. (B) Total number of CD4+ T cells in the dermal site of primary (NaLm) or secondary infection (LmLm) in CCR2.CFP-DTR or wild type (WT) littermate controls (generated from CCR2.CFP-DTR-/+ crosses) treated with PBS or DT. Data is from a single experiment employing n = 4–8 ears per group. (C) Direct intracellular staining for the absolute number of IFN-γ+TcRβ+CD4+ cells per ear in mice as described in (B). (TIF) [file ppat.1006479.s008.tif]
